# A Comprehensive Review of the Role of the Latest Minimally Invasive Neurosurgery Techniques and Outcomes for Brain and Spinal Surgeries

**DOI:** 10.7759/cureus.84682

**Published:** 2025-05-23

**Authors:** Simone Laguardia, Alessio Piccioni, Jorge Eduardo Alonso Vera, Ali Muqaddas, Milko Garcés, Sidra Ambreen, Sarmishtha Sharma, Tara Sabzvari

**Affiliations:** 1 Medicine and Surgery, Università degli Studi di Milano-Bicocca, Monza, ITA; 2 Medicine and Surgery, Università degli Studi di Roma "La Sapienza", Rome, ITA; 3 Medicine, Universidad Católica Santiago De Guayaquil, Guayaquil, ECU; 4 Medicine and Surgery, Medipol University, Beykoz, TUR; 5 Medicine, Universidad Peruana de Ciencias Aplicadas, Lima, PER; 6 Medical Education, University of Texas Health Science Center at Houston, Houston, USA; 7 Medicine, Riga Stradins University, Riga, LVA; 8 College of Medicine, McMaster University, Toronto, CAN

**Keywords:** brain and spine, brain surgery, minimally invasive and robotic spine surgery, minimally invasive techniques, new technologies in neurosurgery, outcomes of neurosurgery

## Abstract

Minimally invasive neurosurgical techniques offer reduced surgical trauma, lower complication rates, and faster recovery compared to traditional open procedures. Approaches such as keyhole craniotomy, endoscopic skull base surgery, endovascular neurosurgery, and percutaneous spinal techniques have advanced the management of cranial and spinal pathologies. These methods aim to minimize tissue disruption, blood loss, and hospital stay while maintaining surgical efficacy. This narrative review explores their evolution, applications, and advantages in modern neurosurgery.

## Introduction and background

Minimally invasive neurosurgery has gained popularity in recent years, transforming traditional open surgery through advanced technology. Open surgery often results in longer recovery times, higher complication rates, and prolonged hospital admissions; it typically requires large incisions, tissue removal or retraction, and bone resection, which, while effective, can lead to several complications such as infections or bleeding, ultimately extending recovery periods [1,2]. Minimally invasive techniques, on the other hand, aim to reduce tissue damage by focusing on specific areas, resulting in less pain and faster recovery [3].

At the close of the 20th century, craniotomy remained the standard surgical approach due to limitations in lighting, inadequate visualization techniques, and the lack of specialized neurosurgical instruments. These constraints often necessitated extensive craniotomies, resulting in significant brain retraction, which increased the risk of neurological damage and adversely affected patient outcomes. The keyhole concept emerged as an alternative, utilizing small, linear scalp incisions for various intracranial pathologies, including aneurysms, tumors, and abscesses. This approach proved to be faster, less traumatic, and contributed to quicker recovery times, alongside a reduction in brain swelling.

Traditional open craniotomies presented several challenges, particularly the risk of traction injuries, which could lead to transient or permanent neurological deficits, as well as intracranial hemorrhages (ICHs). In response, efforts were made to reduce brain retraction during surgery, including the adoption of dynamic traction techniques. However, it is through the advent of minimally invasive surgery that significant progress has been made in minimizing iatrogenic complications. This aligns with the concept of minimizing harm, a principle attributed to Hippocrates, and emphasizes achieving optimal surgical efficiency with reduced patient morbidity. The keyhole approach aims to minimize damage to both the target area and surrounding tissues by utilizing smaller surgical routes, resulting in less trauma and a more favorable recovery trajectory.

Initially, keyhole approaches faced challenges such as narrow fields of view and inadequate lighting. Nonetheless, the introduction of neuroendoscopy, coupled with high-intensity illumination, flexible imaging technologies, and intraoperative assistance from devices such as ultrasound, CT, and MRI, has substantially enhanced the surgical field. These innovations have allowed for optimal lesion visualization, facilitating precise navigation through complex anatomical structures while avoiding critical areas, thus minimizing both intracranial and extracranial tissue damage. In addition, these minimally invasive procedures offer superior cosmetic outcomes, contributing to higher patient satisfaction rates.

This review discusses how these techniques have improved the treatment of uncommon types of head injuries, especially those affecting complex or surgically challenging regions. Minimally invasive techniques are promising; although standard methods are effective and precise, they are often associated with greater morbidity. In appropriate cases, minimally invasive approaches can reduce tissue disruption and improve surgical visualization, potentially leading to better functional outcomes. These methods lead to shorter hospitalizations, less postoperative pain, fewer complications, and faster returns to normal activities [3]. This review also explores how these advances affect the patient's quality of life, focusing on new tools, procedures, and technologies that enhance care for complex cases. As technology continues to advance, the benefits of minimally invasive surgery over traditional open procedures are becoming increasingly evident, particularly for treating rare brain conditions such as deep tumors and vascular malformations, where conventional methods may be less effective.

Hybrid technologies, combining artificial intelligence (AI) and robotics, have further advanced minimally invasive neurosurgery. Robotics improves precision in both spinal and cranial surgeries, which often require highly accurate movements. These innovations are transforming spinal surgery, which traditionally involved more invasive approaches. AI-based analysis helps surgeons interpret data in real-time, allowing for personalized treatment decisions. Augmented reality (AR) provides real-time image overlays, improving depth perception and spatial awareness, further enhancing preoperative planning and intraoperative navigation. The integration of AI and robotic technologies is revolutionizing minimally invasive neurosurgery, particularly for interventions requiring high precision. These innovations hold great potential for improving patient care and outcomes by anticipating complications and tailoring treatment strategies. Based on clinical data, case studies, and longitudinal studies, the review also examines patient outcomes and the effectiveness of these techniques in reducing surgical trauma, complications, and recovery times.

Despite their advantages, minimally invasive techniques have limitations. Successful application requires careful patient selection, preoperative preparation, and expert surgical skills. Complications such as intraoperative bleeding or cerebral edema may arise. The limited workspace offered by minimally invasive access, such as a keyhole, may sometimes be insufficient, necessitating conversion to a more extensive craniotomy. Therefore, balancing the benefits and risks for each patient remains essential.

Techniques such as minipterional (MP) craniotomy, supraorbital or subtemporal keyhole, and endoscopic retrosigmoid craniotomies offer viable alternatives to traditional methods. These techniques are also applicable to spinal surgery, where minimally invasive approaches have been increasingly employed to reduce trauma and improve recovery times. Although these techniques offer significant advantages, challenges such as appropriate patient selection and the need for highly qualified surgical skills remain. Technological advances, particularly in AI, robotics, and AR, continue to improve the effectiveness of minimally invasive procedures, shaping the future of neurosurgery, both for cranial and spinal interventions.

## Review

Techniques in brain surgery

Minipterional Craniotomy for Middle Cerebral Artery Aneurysms

Pterional craniotomy exposure is centered in the pterion, a joint of the frontal, parietal, temporal, and sphenoid bones. This provides broad exposure of the frontal and temporal lobes and offers two corridors: the trans-Sylvian and the sub-frontal. The MP approach differs from the standard pterional craniotomy primarily in size and extent of exposure. While both utilize the trans-Sylvian route to access vascular lesions, the MP approach involves a smaller bone flap, reduced dissection of the temporalis muscle, and less extensive dural opening. These modifications result in better cosmetic outcomes and reduced soft tissue trauma, particularly minimizing the risk of temporalis atrophy and postoperative mastication discomfort. However, the standard pterional craniotomy offers a wider operative field, allowing access to deeper or more complex lesions, and provides greater instrument maneuverability, especially in cases involving large or multilobulated aneurysms or skull base tumors.

The keyhole approaches were introduced by Axel Perneczky, based on the principle of minimizing invasiveness in cranial surgery. These techniques aim to reach the same surgical targets as standard craniotomies while offering better cosmetic outcomes [4,5] and reducing trauma related to craniotomy, brain retraction, and cortical exposure [6]. This, in turn, helps to decrease morbidity and shorten hospitalization time. In modern neurosurgery, the supraorbital (SO) keyhole and MP approaches, the latter introduced by Figueiredo, are widely used for clipping anterior circulation brain aneurysms as less invasive alternatives to the traditional frontotemporal or pterional approaches [7].

The cosmetic benefits of SO and MP are the decrease in the rates of injuring the facial nerve when doing the temporal muscle dissection and avoiding unnecessary dural exposure; other benefits that less invasive approaches can offer are the diminution of postoperative headaches and mastication problems [7,8]. MP can be used for treating aneurysms of the anterior brain circulation as it can reach the anterior and middle cerebral fossa; the vascular targets can include the middle cerebral artery (MCA), anterior communicating artery, and internal carotid aneurysms, and it also has shown promising results for clipping posterior communicating artery aneurysms, with a relatively effective and safe clipping rate across in a meta-analysis published in 2024 by Brown et al. [8]. It also allows extensive surgical access overall, including the entire ipsilateral and medial contralateral anterior cranial fossa, the ipsilateral Sylvian fissure, the medial part of the middle cranial fossa, the anterior part of the midbrain, and the parasellar region [9]. It is a safe and effective approach for clipping MCA aneurysms adjacent to the pterion [10]. Its anatomical targets are restricted to the ipsilateral middle cranial fossa, the Sylvian fissure, the lateral parasellar region, and the posterior section of the anterior cranial fossa [9].

The main limitation of smaller craniotomies or keyholes is that they offer less instrument maneuverability and require exhaustive preoperative planning, considering the importance of the head rotation according to the operative target [9]. Another limitation is the difficulty in managing intraoperative aneurysm ruptures, which may necessitate enlarging the keyhole/craniotomy. Moreover, less invasive craniotomies expose fewer anatomical structures and offer a significantly narrower surgical corridor, potentially leading to complications such as hydrocephalus, hematomas, adhesions, and even intraoperative rupture. Intraoperative bleeding is considerably higher in the suprabrow craniotomy compared to the standard approach. However, it has been shown to be a safer option for unruptured MCA aneurysms, with a lower rate of intraoperative rupture. This is due to the exposure of the medial Sylvian fissure upon frontal lobe retraction, allowing proximal vascular control. A further advantage of the MP approach is that it offers a wider view of MCA aneurysms and lesions near the interpeduncular region [1].

As described, MP has better cosmetic outcomes and offers the same main anatomical corridor, which is the trans-Sylvian; on the other hand, it gives a narrow workspace for the surgeon, and it can lead to more complications for treating big-size lesions, but in vascular surgery, it is safe and effective for clipping anterior circulation aneurysms. By contrast, the classic pterional craniotomy can be tailored to use the sub-frontal corridor and give more anatomical space for treating skull base lesions. The election of the craniotomy must be taken by considering the patient lesion, which can lead to better postoperative outcomes.

Supraorbital Keyhole for Aneurysms and Tumors

The SO approach provides access to the lateral sub-frontal corridor, minimizing temporal lobe exposure and avoiding the trans-Sylvian corridor. It is an alternative to the standard frontotemporal craniotomy, featuring a shorter, eyebrow skin incision and a smaller craniotomy, improving cosmetic outcomes. This approach also avoids the extension of the temporal muscle, reducing the risk of postoperative complications such as temporomandibular joint (TMJ) problems, chewing difficulties, mouth-opening limitations, and late muscle atrophy, thereby reducing hospital stay.

The SO approach provides access to the entire ipsilateral and medial contralateral anterior cranial fossa, the parasellar region, the ipsilateral Sylvian fissure, the medial part of the middle cranial fossa, the anterior midbrain, the optic chiasm, and both optic nerves from the anterior perspective [5,7]. It is effective for treating anterior circulation aneurysms, including those of the anterior cerebral artery, proximal MCA, supraclinoid, paraclinoid, posterior communicating, and anterior communicating aneurysms. It can also be extended by proximal dissection of the Sylvian fissure, providing access to the carotid-oculomotor triangle and exposing the internal carotid artery. In addition, SO can be used for clipping contralateral paraclinoid aneurysms.

The SO approach is also highly effective for treating midline tumors such as craniopharyngiomas and meningiomas, in addition to aneurysms. In these cases, the approach provides excellent access to lesions located in the anterior cranial fossa, ventral portion of the middle fossa, and the parasellar region. Furthermore, the SO approach can be combined with an orbitotomy to expand the sub-frontal corridor, which is particularly useful for accessing lesions like tuberculum meningiomas.

Despite its many advantages, the SO approach has some limitations. The size of the craniotomy may be inadequate for accessing pathologies that exceed the margins of the approach. It can also be complicated by brain swelling in cases that require more extensive exposure, such as glioblastomas. In vascular neurosurgery, SO is widely used for clipping unruptured aneurysms, while ruptured aneurysms may be better approached through standard craniotomies to avoid complications due to brain swelling. In addition, the smaller craniotomy may limit visual access to high-positioned basilar apex aneurysms, which require larger craniotomies for adequate exposure.

As with any surgical approach, the SO approach carries risks, including potential injury to the SO nerve, which can lead to sensory disturbances, ptosis, and other complications, such as frontal nerve injury. These risks can be minimized with careful surgical planning and technique. However, when appropriately selected, the SO approach offers a less invasive alternative to standard craniotomy, with deep access, excellent maneuverability, better cosmetic results, and minimal approach-related morbidity, making it suitable for a wide range of patients.

Endoscopic Retrosigmoid Approach for Deeper Structures

Retrosigmoid craniotomy, or lateral suboccipital approach, is one of the most used approaches for the posterior fossa in neurosurgery. It is safe and efficient and offers an anatomical view of the cerebellopontine angle, foramen magnum, and petroclival regions [11]. The craniotomy is based on the asterion, the central landmark. It is located at the junction of the lambdoid, occipitomastoid, and parietomastoid sutures, and it is an anatomical landmark for identifying the transverse sigmoid junction.

The advantage of using endoscopy in the retrosigmoid approach lies in its ability to enhance the visualization of anatomical structures. In the superior neurovascular complex, the endoscope provides detailed views of the superior petrosal vein (SPV), the trochlear nerve, the proximal segments of the superior cerebellar artery (SCA) and posterior cerebral artery (PCA), the porus trigeminus, the trigeminal root entry zone at the mid-pons, the origin of the ipsilateral oculomotor nerve within the interpeduncular fossa, and the bifurcation of the basilar artery. For the middle neurovascular complex, it significantly improves visualization of the facial-vestibulocochlear nerve complex at its junction with the brainstem, the internal acoustic meatus, the labyrinthine and subarcuate arteries, the anterior inferior cerebellar artery (AICA) and its anatomical relationship with the facial-vestibulocochlear nerve complex, the abducens nerve, the origin of the facial nerve as it courses anteriorly and parallel to the vestibulocochlear nerve, the inferior olive, and the terminal branches of the AICA. In addition, cranial nerves V, VII, VIII, IX, and X are observed passing through their respective skull base foramina. In the inferior neurovascular complex, the endoscope provides superior visualization of the dorsal surfaces of the glossopharyngeal and vagus nerves, the hypoglossal nerve rootlets, the posterior inferior cerebellar artery (PICA), the lower cranial nerve rootlets, and the bony exits of cranial nerves IX, X, and XII [12].

Endoscopy-assisted microvascular decompression offers several advantages over microscopic vascular decompression. It is associated with smaller surgical incisions and shorter operation times. Its ability to provide a broader and more precise view of the entire surgical field enables more accurate identification of blood vessels that cause vascular conflict, reducing the risk of incomplete treatment or insufficient decompression. By contrast, the microscope's limited field of view requires frequent adjustments during surgery about the position and focal length, making assessing the overall nerve and vascular anatomy more challenging. In addition, it minimizes surgical trauma, both external and internal, and contributes to lower recurrence and complication rates. However, using an endoscope requires advanced technical proficiency, as it occupies part of the surgical space. Despite this learning curve, the enhanced visualization and precision offered by endoscopy make it a superior tool for microvascular decompression in many cases [13].

Endaural Subtemporal Keyhole Approach to the Middle Cranial Fossa

Past surgical approaches: At the end of the 20th century, craniotomy remained a standard approach in many neurosurgical procedures due to limitations in lighting, visualization, imaging accuracy, and the lack of specialized instruments. These constraints often forced surgeons to adopt extensive approaches with significant brain traction, increasing the risk of neurological damage and compromising patient outcomes. In 1971, D. Wilson introduced the keyhole concept, using small linear scalp incisions to access pathologies such as aneurysms, tumors, and abscesses [5]. This technique proved to be simpler, quicker, and less traumatic, leading to faster recovery and reduced brain swelling. Open and extended craniotomies were associated with complications such as traction injuries, which could result in transient or permanent deficits, and even large intracerebral hemorrhages (ICHs). Although techniques like dynamic traction were introduced to mitigate these effects, none matched the minimal invasiveness offered by keyhole surgery, where reducing iatrogenic injury and maximizing efficiency are central goals.

Minimally invasive surgery (MIS) aims to avoid unnecessary damage to both intracranial and extracranial tissues, reducing trauma to healthy structures and enhancing recovery. The smaller the surgical corridor, the fewer functional and structural elements are put at risk. The keyhole approach, supported by modern technologies, allows for safe and effective procedures with fewer complications and faster rehabilitation. Early limitations, such as narrow fields and poor illumination, were overcome by the development of neuroendoscopy, high-intensity lighting, flexible instrumentation, and image-guided systems like intraoperative ultrasound (US) and MRI. These tools enhance lesion visualization, minimize brain retraction, and reduce neurological complications. Keyhole entry points are selected for minimal trauma, optimal access to deep targets, and favorable cosmetic outcomes. Today, various keyhole techniques are available, including the SO eyebrow arch, SO lateral, intraorbital, mini orbitozygomatic, lesser pterygoid, and anterior longitudinal fissure approaches.

Presentation of the latest keyhole: In recent months, a new keyhole has been presented, called the subtemporal endaural, EAST, which combines an incision hidden by the skin folds of the ear, providing excellent cosmetics, and a craniotomy of the keyhole along the floor of the lateral central fossa, allowing surgeons to access both superficial and deep lesions in the temporal lobe or central fossa. A study conducted in California in 2024 described this approach in six patients, three of whom were operated on due to a temporal lobe encephalocele and dehiscence of the tegmen tympani with CSF leak, two had a basal ganglia hematoma, and the last had a resection of an intra-axial temporal lobe tumor [14]. The surgery was performed under general anesthesia; the patient, supine, had his head facing the contralateral side of the pathology. The patient could be placed on a gel headrest or fixed in a Mayfield's head clamp to stabilize it. The preparation included betadine on the surgical site and the ear canal, which was packed with gel foam to prevent the risk of fluid ingress during surgery. The first phase of the surgery was a skin incision that began above the earlobe and transected the tragal cartilage, the tip of which was retracted and reflected anteriorly. 

To obtain aesthetic closure, it is essential not to remove the tip of the tragal cartilage from the skin flap. The incision was placed posterior to the superficial temporal artery, reducing the likelihood of arterial lesions compared to the standard pretragal incision despite the small risk of TMJ injuries located underneath. The incision length could be modified depending on the trajectory required for the pathology and based on different anatomical variants. After the incision, zygoma root dissection was performed to provide exposure to the middle fossa floor, and the tragal cartilage served as a reference point for safe exposure. A sharp dissection along the ventral aspect of the cartilage minimized the risk of entering the parotid gland and injuring the frontal branch of the facial nerve, a complication more commonly seen with standard pretragal incisions. The lateral temporomandibular ligament, which attaches to the zygoma, must be elevated and reflected inferiorly to properly expose the root of the zygoma. 

Additional exposure of the soft tissues was achieved using periosteal elevators and monopolar cautery, while retraction was maintained with a small Weitlaner retractor or fish hooks. It is essential to avoid resection of the glenoid fossa or upper articular surface of the TMJ, as this could result in malocclusion, limited jaw opening, and postoperative pain. Once adequate exposure is achieved, a burr hole is created at the root of the zygoma to access the dura mater, which may either be elevated or opened depending on the surgical objective. Closure of the craniotomy includes placement of a burr hole cover, absorbable sutures for the deeper layers, and simple interrupted stitches for the final tragal portion of the incision. In this study and others, one involving 14 patients with cerebrospinal fluid (CSF) leak and tegmen defect, and another involving five patients, the EAST approach provided sufficient exposure to address the pathology. No injuries or surgical complications were reported, resulting in reduced hospital stay, lower morbidity, shorter operative times, and a higher number of treated cases [15,16]. Even after a 20-month follow-up, no patient showed signs of recurrent CSF leakage or other complications.

Three cases of women operated with the EAST approach

The first case was a 65-year-old woman with obesity and unilateral otorrhea. She was diagnosed with temporal bone encephalocele and CSF leak, and she had no history of trauma or surgery in the area. The CT scan showed focal dehiscence in the left tegmen tympani and fluid in the mastoid cells, and the MRI confirmed the presence of the fluid in the left temporal bone. The EAST approach was used for encephalocele resection and CSF leak repair; during surgery, a 20 x 15 mm burr hole was made above the zygoma to access the central fossa floor. After the identification and resection of the encephalocele, the repair for the dura was done, with a dural graft inlay, a primary closure of the dural defect, a fascial graft on the dehiscence of the tegmen, and then the application of fibrin glue. A titanium mesh plate was used to cover the burr hole. The patient was discharged on postoperative day 2 without complications, and then, in the three-month follow-up, she had no recurrence of CSF leakage [14]. 

The second case was a 47-year-old woman with hypertension, diabetes, and obesity who presented to the emergency department with altered mental status and left-side weakness. She had a BP of 243 mmHg, and a head CT showed a hemorrhage of 5 x 5 x 4 cm in the right basal ganglia. For the ICH evacuation, the EAST approach was used, and with the help of neuronavigation and a Mayfield head clamp, precise intra-axial targeting was realized. The 15 mm burr hole was used to insert a 12 mm cylindrical port, which allows the use of an endoscope for the evacuation of the hematoma. A DuraMatrix inlay and DuraSeal tissue sealant was used to reduce the risk of CSF leak, and then a titanium cover was placed to cover the burr hole. Postoperatively, the patient had improved consciousness and had no residual hematoma; she was discharged to rehabilitation without complications at the follow-up three months later [14].

The last case was that of a 58-year-old woman who presented to the emergency room with dizziness and headache; the MRI showed a tumor of the inferior temporal gyrus. During surgery, neuronavigation was used to evaluate the exact size and position of the burr hole needed. Once the dura was opened with microscopic assistance, the tumor was circumferentially dissected using a bipolar suction technique and subsequently resected with the standard microsurgical procedure. The postoperative MRI showed no evidence of complications, and after a year, the patient had no complications [14].

Other studies and surgeries

In 2023, a study analyzed three patients treated for idiopathic temporal bone encephalocele with CSF leak via the EAST approach; each presented a unilateral tegmen tympani defect with CSF otorrhoea. The incision used in the surgical intervention was made from the anterior-superior edge of the tragus, progressing in a superior-posterior way along the upper edge of the helix. A rolled sheet of Dura Matrix was placed under the temporal lobe to cover the dural defects. All patients undergoing the operation were discharged in two to three days, the postoperative CT showed no complications, and all had the resolution of CSF leaks without recurrence [17]. In 2020, 14 patients were treated for CSF otorrhoea, with a mean age of 60.7 years and an average BMI of 32.8, through the EAST approach. During the operation, no one had complications, with a mean operation time of 103 minutes, an average hospital stay of 1.4 days, and no complications on the follow-up 20.3 months later [15]. Another patient, a 57-year-old male, was operated on in 2017 for severe meningitis, a spontaneous leakage of CSF due to bone defects in the tegmen tympani. In the surgery, after creating the subtemporal keyhole, a 30-degree endoscope was used to inspect and repair the defects of the tegmen tympani. The patient had a very smooth recovery, with no recurrence of CSF leaks, mastication issues, or pain [15]. Moreover, in 2019, two patients with osteoblastoma and granuloma received a subtotal resection, and three others with cholesteatoma, cholesterol granuloma, and schwannoma had a total resection. None of them suffered from particular complications except one who had cerebral edema, but no progression was found in him during the follow-up [18].

Prospects and limitations

This new subtemporal approach to the keyhole could provide adequate removal for several pathologies and specific tumors of the middle cranial fossa, which were considered very challenging due to the complex anatomy and the critical neurovascular structure. The combination of endoscopy and this keyhole technique also helps minimize complications, provide perfect visualization with a minimally invasive route, and avoid temporal lobe retraction. Indeed, the approaches to the keyhole are not adequate for every type of operation; the patient's choice must be careful; it is necessary to take into account the size and nature of the various pathological lesions, the conditions of the brain tissue near the injury and the different risk factors that could increase complications during the operation. A cadaveric study also proposes this approach to repair the dehiscence of the semicircular canal, and indeed, further studies are needed to verify if it is possible and feasible in vivo and correlated with an improved outcome [19]. More extensive randomized control trials are also required to verify and confirm the long-term outcome of this EAST keyhole approach.

Endoscopic approaches for skull base malignancies

Endoscopic skull base surgery started as a modified view for treating simple intrasellar pituitary adenomas. However, a recent revolution in the surgical field has allowed this procedure to be used for middle, anterior, and posterior fossa skull base lesions [20]. Since the description and implementation of the nasoseptal flap reconstruction by Hadad et al., the decrease in infection and CSF leakage made the endoscopic trans-sphenoidal approach a real alternative for pituitary malignancies when compared to more invasive transcranial techniques [21]. From that point forward, through multiple cost-benefit analyses and international publications, there has been a significant improvement in this surgical approach, allowing it to be used to explore various brain lesions in the skull base. The endoscope's minimally invasive nature and enhanced field of view allowed its applications to expand while replacing traditional surgical techniques with poorer clinical outcomes [22].

Pituitary adenomas have been the hotspot of endoscopic brain surgery, arguably from the day it was introduced. The anatomical location of the pituitary gland makes it an attractive site to reach endoscopically, especially when compared to the possible postoperative complications a craniofacial approach poses [23]. Compared to microscopic transsphenoidal or open transcranial approaches, improvements in surgical field illumination, visualization, and minimal retraction make the endoscopic approach an excellent alternative when dealing with large or giant pituitary adenomas, especially if there is not significant lateral extension. 

Endonasal approaches to these types of lesions have a higher rate of gross total resection, improved field of vision, and a lower grade of recurrences. Regarding post-surgical complications, CSF leaks and meningitis rates also occur less frequently in endoscopic approaches [23]. Given the nature and physiology of the pituitary gland, complications from endoscopic pituitary surgery can be categorized into two main types: surgical and endocrine-related complications, affecting either the anterior or posterior lobe. The two main surgical complications seen in endoscopic pituitary surgery are meningitis, seen in 1-2% of cases, and CSF leaks, seen in 3-5% of cases, whereas the main endocrine-related complications are hypopituitarism, seen in 8-16% of cases, and diabetes insipidus seen in <1% of cases; however, it tends to resolve sporadically in most cases [24]. Although rare, more severe surgical complications involve injury of major vessels, especially to the internal carotid arteries; however, the incidence is too small to be considered, with rates as low as 0.68% [22]. It is also essential to take into consideration that surgical pituitary tumors require a multidisciplinary approach to reach optimum recovery and minimize recurrence rates. Up to 10% of pituitary adenomas cannot be managed only with a trans-sellar approach, usually requiring additional bone removal, neurovascular transposition, and medical treatment with or without radiation therapy to reach long-term tumor control [25,26].

Depending on the literature, craniopharyngiomas may or may not be considered skull base tumors as they originate from the pituitary stalk. However, their importance must be discussed due to the anatomical similarity and surgical approach. Similar to pituitary adenomas, the endoscopic approach was not considered in the pre-vascularized flap era due to the postoperative risks it posed. However, the minimally invasive nature of the trans-sphenoidal surgical corridor makes it a valid candidate for lesions extending past the sella turcica. The degree of retraction in any neurological procedure is often directly correlated with the neurological function of the area intervened. Therefore, an endoscope's minimal retraction is usually seen as the beneficiary for complex procedures to preserve neurological function. Selection of the most suitable approach must be planned thoroughly, especially if the lesion is adherent to neurovascular structures like the pituitary stalk or optic chiasm [27].

In most cases, the endoscopic approach has been elected when dealing with midline suprasellar and retrochiasmatic craniopharyngiomas. However, compared to a transcranial approach, this approach lacks maneuverability and surgical visibility when resecting larger lesions or lesions extending laterally toward the internal carotid artery [28]. The third ventricle invasion is also a determining factor for choosing the right surgical approach. These tumors can typically be approached with either method [29].

Meningiomas are the most common primary intracranial tumors. Their treatment is highly variable and depends entirely on the location, size, and invasiveness of the cancer. In an ideal scenario, the best surgical approach would have a complete tumor resection with minimal to no brain retraction. The benefits of an endoscopic approach compared to a transcranial approach are having direct access to the dural portion of the tumor, better exposure of midline skull base tumors, identification of the internal carotid arteries and their deep or complex vascular supply, better optic nerve decompression, better cosmetic outcomes, and reduced brain manipulation [30]. Over the years, the increased use of endoscopic approaches in the anterior and posterior fossa has made it the most optimum treatment for meningiomas located in the midline of the skull base. Moreover, it is a perfect alternative when dealing with small- to medium-sized meningiomas in the anterior fossa, like tuberculum sellae or planum sphenoidal meningiomas, due to its low degree of brain retraction [31]. 

However, among the common complications of anterior fossa surgery, using a transplanum-transcribiform approach can result on olfactory nerve damage, leading to olfactory disturbance. However, integrating a transcranial and transnasal approach reduces the risk of anosmia [32]. The endoscopic approach has recently been considered an alternative to transcranial approaches for the petroclival region, including primary tumors from this region (petroclival meningiomas, chondrosarcomas), as well as those that invade the petroclival area from distant regions (T4 sinonasal tumors, trigeminal schwannomas). Regardless of the nature of the cancer in the petroclival region, the location and invasion of close neurovascular structures, as well as its deep paramedian location, determine the choice of the surgical approach. Compared to a transcranial approach, endoscopic transsphenoidal surgery offers better illumination and visualization of the surgical field. This entry point can be used for an anterior petrosectomy or a transcavernous approach. The endoscopic approach does lack maneuverability and space when approaching petroclival tumors that extend laterally, behind the sixth cranial nerve [33].

Surgery has been a highly effective and potentially curative treatment for pituitary tumors ever since the first operating microscope was introduced in the early 1960s. Microscopic surgery was kept as the first line for pituitary surgery until the introduction of the endoscope in the early 1990s [34]. The endoscopic approach opened doors to new surgical techniques, especially in neurosurgery, and created a lot of interest due to the low grade of brain retraction and improved visual field during procedures. The anatomical site of the pituitary gland mainly makes it a perfect scenario in most cases for an endonasal trans-sphenoidal approach due to the natural canal the endoscope has to travel and the proximity from the nasopharynx to the gland. It has a minimally invasive nature and tends to have an increased average volumetric resection of tumors when compared to microscopic surgery. However, multiple variables tend to be considered when deciding on a surgical approach, such as tumor size and involvement in neurovascular structures like the optic chiasm or internal carotid arteries [35]. Moreover, endoscopic surgery has an increased overall cost and operating time now that an otolaryngologist must do a nasoseptal flap to prevent surgical complications like CSF leakage or meningitis [36].

Despite the technique used in the pituitary gland, the surgical risks tend to be very low, while the surgeries are both practical and safe. There are many ways to compare the advantages and disadvantages of endoscopic versus microscopic pituitary surgery. However, the data do not support one over the other [37]. Microscopic surgery tends to have a decreased volumetric resection of pituitary tumors. However, it also has a reduced operating time and an average of 16% less operational costs [38]. Endoscopic surgery, on the other hand, demonstrates an increased volumetric resection, which leads to a decrease in postoperative secondary treatments (like radiation therapy) and fewer cases of second surgeries. It does, however, have a higher cost and operating time. There has been a national trend of transition from microscopic to endoscopic pituitary surgery recently, but in experienced hands, both alternatives should give excellent results [39].

Technological advances have been made to improve outcomes and minimize human error in endoscopic pituitary surgery. The NeuRoboScope is a perfect example of this, as it is a revolutionary device that acts as a third hand in minimally invasive surgeries by holding the endoscope to allow the primary surgeon's active use of both hands. Besides this, its system includes a human-robot interface that regulates and facilitates the interaction between the primary surgeon and the robot assistant. It manages to reduce the surgeon's workload during the surgery while increasing efficiency and shortening the average operating times [40].

Role of endovascular procedures in vascular neurosurgery

Endovascular methods have gained prominence in vascular neurosurgery, providing less invasive options than traditional surgical approaches for aneurysms and arteriovenous malformations (AVMs). Such techniques, especially in high-risk cases, provide better recovery and fewer complications, improving patient outcomes. Aneurysms are managed endovascularly, with microsurgical clipping or a hybrid of both; treatment options are based on the patient's anatomy. Recent investigations reveal the benefits of endovascular therapy (e.g., coiling) in dramatically improving quality of life and reducing complications such as intracranial infections and postoperative deficits. However, rebleeding continues to be a challenge. Moreover, the preoperative embolization of AVMs leads to less intraoperative bleeding and lowers the surgical risk. Endovascular approaches are set to become progressively more influential in determining outcomes in vascular neurosurgery. 

As previously mentioned, sometimes aneurysms can be treated through endovascular procedures, microsurgical clipping, or a combination. The decision to select one approach over the other remains debatable. Ultimately, the treatment choice should be individualized, carefully considering the patient's clinical condition and determining which option offers the most significant benefit. A meta-analysis conducted by Zhang et al. in 2023 demonstrated that patients with MCA aneurysms treated with interventional embolization experienced better quality of life and a lower incidence of intracranial infections. By contrast, surgical clipping demonstrated advantages such as lower rates of residual aneurysm neck, recurrence, and rebleeding. There was no significant difference in the occurrence of cerebral vasospasm or ischemic stroke between the two treatment modalities [41].

Meta-analysis has shown several roles of endovascular procedures in modern neurosurgery. A work made by Pen et al. in 2022 concluded that in the MCA, aneurysms treated by coiling reduced the risk of postoperative complications like neurological deficits, vasospasm, and cerebral infarction, in comparison with clipping, but also demonstrated that coiling was associated with more risk of rebleeding, hydrocephalus, and rate of mortality and lower rate of complete aneurysm occlusion [42]. Posterior circulation aneurysms are more challenging for microsurgical clipping due to the anatomical locations and the complexity of the craniotomy. A meta-analysis published by Fotakopoulus et al. in 2021 demonstrated that endovascular coiling can be considered a good alternative, as it offers less postoperative complications like the treatment of anterior circulation aneurysms, as mentioned before, but the problem remains in that there can be no complete occlusion of the aneurysm and reintervention can be done [43]. AVMs are very challenging entities in neurosurgery; they require complex anatomical knowledge and technique to reach better outcomes. Endovascular procedures in AVMs offer many advantages and are used with microsurgical resection by reducing AVM's blood supply and size, minimizing operative bleeding. A retrospective study made by Alfter et al. in 2023 established that preoperative embolization in the management of high-grade AVMs reduced the size and vascularity of the AVM and also facilitated safer and more effective microsurgical resection. The procedure helps by occluding deep arterial feeders and lowering intranidal flow, thus minimizing surgical complications [44]. Another study made by Izumo et al. in 2022 also demonstrated that preoperative embolization with Onyx effectively reduces intraoperative bleeding and may slightly shorten surgery duration [45].

The role of endovascular procedures in modern neurosurgery has shown improvement in patient outcomes by offering, in some circumstances, the same results as open surgeries. Still, it also leads to complications such as increased rebleeding rates. However, the tendency is to manage patients by multidisciplinary approaches to give better results; endovascular procedures can serve as coadjutant treatments and can be combined with open brain surgeries. In addition, every patient must evaluate the treatment selection, considering the pathologies they have and the risks or benefits of the intervention.

Table 1 summarizes and compares the different minimally invasive brain surgery techniques mentioned above with open surgery.

**Table 1:** Summary of the newest minimally invasive neurosurgery techniques for brain surgery

Techniques	Applications	Advantages	Risks	Outcome vs. open surgery
Mini-pterional craniotomy	• middle cerebral artery aneurysms	• reduced dural exposure and brain retraction • less tissue damage	• limited control during aneurysms rupture • hydrocephalus or hematoma from corridor collapse	• faster recovery • reduced post-operative complications
Supraorbital keyhole approach	• anterior communicating artery aneurysms • dural-based tumors in the anterior and middle fossa • carotid-oculomotor triangle’s lesions	• aesthetic incisions and minimal muscle dissection • broad access to the anterior and middle fossa	• limited access to deeper or larger pathologies	• faster recovery • lower complication rates and morbidity • improved cosmetic results
Endaural subtemporal keyhole (EAST)	• temporal bone encephalocele • basal ganglia hematoma • intra-axial tumors of temporal lobe	• excellent access to middle fossa • minimal lobe retraction • hidden incisions	• injury to temporomandibular joint • potential complications due to anatomical variants	• faster recovery • lower complication rates and morbidity • improved cosmetic results
Endoscopic retrosigmoid approach	• neurovascular conflicts and posterior fossa tumors	• reduced surgery time • superior visualization using endoscopy	• required advanced skills due to smaller corridors	• faster recovery • lower complication rates • reduced surgical trauma
Endoscopic skull base surgery	• pituitary adenomas • craniopharyngiomas • petroclival tumors • meningiomas	• better access to deep-seated structures • reduced brain retraction	• incomplete resection • CSF leaks • hypopituitarism • meningitis	• faster recovery • lower complication rates and morbidity • improved cosmetic results
Endoscopic pituitary surgeries	• pituitary adenomas • sellar lesions	• enhanced visualization • lower recurrence	• higher costs • CSF leaks or meningitis	• reduced secondary treatments

Techniques in spine surgery

Endoscopic Keyhole Approach for Minimally Invasive Spinal Surgery

Over the past 40 years, the endoscopic keyhole technique for minimally invasive spine surgery has advanced rapidly as an alternative to traditional spinal procedures for neurodegenerative diseases. It preserves the conventional neurosurgical approach while minimizing the surgical corridor and producing the best results. The primary purpose of an endoscope in minimally invasive neurosurgery is to make surgery more visually accessible and avoid accidental dissemination, preventing surgical injuries and thus lowering the rate of morbidity. Compared to uniportal, biportal endoscopic spinal operations are more practical and commonly performed. The surgeon's choice and the clinical circumstance determine preference [46]. The transforaminal and interlaminar methods are the two most used techniques for endoscopic spine surgery. A small incision is made to insert the endoscope, preserving the normal functioning of the surgical site. Despite its primary usage in minimally invasive lumbar spine surgery, endoscopy has gained popularity in treating thoracic disc herniations and intrathoracic non-spinal lesions and correcting spinal abnormalities [47].

Endoscopic spinal surgeries, in comparison to other endoscopic surgeries of the body, present unique challenges. The lesions are often far from the skin and absent an open space. Usually, extensive bone work complicates the surgery, and preserving the neural tissue becomes challenging. In such cases, an endoscope aids the surgeon by allowing a detailed inspection of the surgical site, thus preventing surgical complications due to injuries [48]. Various other spinal pathologies can be treated by endoscopic MIS, such as cervical spondylosis, spinal tumors, meningiomas, paracentral and extraforaminal disc herniations, spinal stenosis, cervical epidural abscess, cervical radiculopathy, spinal infections such as infectious spondylodiscitis, and many other neurodegenerative diseases.

A retrospective study was conducted by Feng et al. from May 2021 and September 2023 on 63 cases that were treated for cervical pain and disability caused by spondylotic radiculopathy. Preoperative baseline values and postoperative outcomes were compared after the data were statistically arranged and compared. Oblique spinal endoscopy (OSE) keyhole excision of the posterior cervical nucleus pulposus is less intrusive and promotes better postoperative recovery, making it a favorable clinical alternative. There are limitations in the perception of results due to its retrospective design and cohort follow-up, but the relevant data collections continue [49,50].

Lv et al. conducted a systematic review. Included were 14 observational studies of cervical spondylosis with 479 patients, 197 myelopathy cases, and 207 radiculopathy cases. The study backs up Feng et al.'s findings and demonstrates that posterior MIS is a safe and efficient way to treat cervical spondylosis [51].

Between December 2015 and October 2018, Liu et al. treated 29 patients for nucleus pulposus extirpation and percutaneous endoscopic posterior cervical keyhole fenestration decompression [51]. Thirteen females and 16 males, ages 39 to 78, with an average age of 49.7 years, were treated. The herniation occurred in multiple cervical regions, ranging from C1 to C7. The duration of the procedure, intraoperative blood loss, hospitalization, and complications were noted and documented. Following surgery, the patients were monitored for an average of 19.4 months, ranging from 11 to 43 months. According to the Macnab criterion, the effectiveness was judged as exceptional in 11 cases, good in 15 cases, and evil in two instances at the most recent follow-up, with a recovery rate of 89.7%. The advantages of percutaneous endoscopic posterior cervical keyhole fenestration decompression and nucleus pulposus extirpation include MIS that is safe and effective, rapid recovery, and satisfactory effectiveness for paracentral cervical disc herniation with simultaneous compression of nerve roots and spinal cord [51].

In a comprehensive review of endoscopic spine surgery by Jitpakdee et al., 312 articles were published between January 1989 and November 2022. It was concluded that there had been a shift from the uniportal approach to the biportal approach in recent years due to the wide-ranging visuals available for portal endoscopic surgery. The two main approaches for endoscopic spine surgery are the transforaminal and interlaminar approaches. Biportal endoscopic spinal surgeries are more reasonable than uniportal and can be performed broadly. Preference varies according to the clinical situation and surgeon’s choice [46]. It is simple to approach, has more accuracy, and is favorable for interbody fusion. The uniportal approach provides a minor corridor for tissue dissection [46,52].

In a retrospective observational study by Lin et al., 508 patients were treated with spinal endoscopic surgery, of which 60 patients were treated for infectious spondylodiscitis from October 2006 to March 2017. All these 60 patients were screened with enhanced magnetic resonance imaging and plain film radiography of the affected region to obtain clear evidence of the infection. All patients reported rapid pain relief post-operation and antibiotic therapy. Causative agents for 34 patients were identified, and only two patients underwent infectious relapse and were treated with a second round of endoscopic surgery. The infection did not appear again in any of the treated patients. No surgery-related complications were seen in any of these patients post-endoscopic keyhole surgery for the infectious spondylodiscitis, and no traditional surgical intervention was required later. After examining the culture rate, recurrence rate, kyphotic change, and postoperative problems associated with the procedure, it was determined that this new endoscopic procedure is safe and effective and may even represent a new trend in treating similar disorders [53].

In a systematic review by Verdú-Lopez et al., it has been noted that the incidence of spinal infections is increasing. Various spontaneous and iatrogenic pyogenic and non-pyogenic spine infections can be treated differently based on the infection's location and extent. Eradicating infection, protecting neurological function, and maintaining structural integrity are the three main goals for treating spine infections. The minimally invasive surgeries with the aid of endoscope and image-guided paths carved a new path for minimal invasion while performing surgery with higher accuracy and recovery rate [54].

Two hundred forty-five intradural tumors were surgically treated in a retrospective analysis by Mende et al. from 2009 to 2016. One hundred fifty-one of these lesions were either neuromas (n = 72) or intradural extramedullary meningiomas (n = 79). Dumbbell neuromas accounted for nine (12.5%) of the neuromas. There were 94 intramedullary tumors; 53.9% of the patients were female, and their average age was 51.4 years. The follow-up period was 46.0 months on average. Although a mini-open keyhole technique could resect all meningiomas and neuromas, it could only access 5.3% of the intramedullary lesions. Then, 10.6% needed a two-level laminectomy, 7.4% needed a hemilaminectomy, and 76.6% needed a laminotomy, out of the 94 patients with intramedullary tumors. During follow-up, only two of the patients with intramedullary tumors required stabilization due to increasing cervical kyphosis. Following surgery using a mini-open (keyhole/interlaminar) technique, none of the other patients experienced any spinal problems. Compared to patients treated with the keyhole minimally invasive method, there were substantially higher surgery-associated problems within the large exposure window (19.1% vs. 9.6%, p < 0.01). A keyhole interlaminar technique can safely resect intradural extramedullary and intramedullary diseases. While still enabling full excision of these tumors, avoiding laminectomy, laminotomy, and even hemilaminectomy maintains spinal stability and dramatically lowers comorbidities. Considering macroscopically complete resection rates of 100% in our meningioma cohort and 98.4% in their neuroma cohort, the cohort shows that resection of intradural diseases with an interlaminar technique is a viable alternative [55].

The above data suggest the advantages of the keyhole approach in spinal pathologies. Using an endoscope in the keyhole approach of minimally invasive surgeries has led to better preservation of the normal physiology of the surgical site. It has the least invasive window to the surgical site and unparalleled visualization. It decreases surgical mistakes and injuries, decreasing comorbidities. It has remarkably shown better cosmetic outcomes, faster recovery rates, and accurate surgery performance. The outcomes depend on preoperative values and are determined by the complications rendering mid-surgery due to the phase shift. A real-time imaging technique can help avoid them; soon, science will overcome these hindrances.

Percutaneous Endoscopic Lumbar Discectomy (PELD) for Sciatica and Disc Herniations

Percutaneous endoscopic lumbar discectomy (PELD) is an innovative technique introduced for treating herniated discs, initially proposed by Dr. Parviz Kambin [56]. Kambin believed the intervertebral foramen was an ideal access route for the endoscopic approach to herniations, delineating the so-called "Kambin's triangle." The nerve root, which constitutes the hypotenuse, the proximal vertebral plate (base), and the intervertebral articular processes (height) delimited this right-angled triangle.

The first percutaneous endoscopic technique, based on the lateral transforaminal approach, is PETD. PETD has two main techniques: the YESS technique, developed by Yeung in 1997, and the TESSYS technique, introduced by Hoogland in 2003. The YESS (inside-out) technique allows the herniated portion to be removed from inside the annulus fibrosus using Rongeur forceps and is particularly indicated for urgent central hernias. Although it can lead to a reduction in the height of the intervertebral disc, Yeung perfected it himself thanks to indigo carmine. This dye allows the damaged portion of the nucleus pulposus to be identified exclusively. This is possible because encountering a lesion of the extracellular matrix, this portion is more permeable to the dye.

On the other hand, the TESSYS (outside-in) technique involves using a drill to remove the herniated portion from the outside. It is then followed by reconstructive plastic surgery. Compared to YESS, TESSYS provides gradual decompression and minimizes manipulation of nerve structures.

PETD is mainly used for extruded and migrated hernias, with compression on the root and dorsal root ganglion, especially at levels up to L3 and L4. However, at lower levels, the transforaminal approach is limited due to the reduced available space; to overcome this, in 2006, Ruetten introduced a second type of PELD, the PEID (interlaminar approach). As you go down a level, the interforaminal space decreases, but the interlaminar space increases. At the levels of L5 and S1, the high iliac crests and the size of the transverse process of L5 can hide the herniated disc, making a transforaminal access complex.

PEID (interlaminar discectomy), introduced by Ruetten in 2006, uses the passage through the interlaminar system, making it particularly suitable for treating difficult and complex hernias. It is used above all for hernias that exert compression on the root emergence and the dural sac, preforaminal and paramedian hernias (especially at the level of L5 and S1), and for highly migrated or calcified hernias, often associated with stenosis of the central and lateral canal. This approach allows easier resection of the ligamentum flavum and the hypertrophic joint, effectively resolving any compression caused by these structures.

Furthermore, a third approach, posterolateral, is indicated for contained hernias or disc protrusions [56]. PEID can be performed with either an inside-out or outside-in approach. However, it has the disadvantage of having to slightly move the dural sac to remove the herniated fragments, resulting in an incidence of neurological complications of 1.1%, especially with the Hoogland TESSYS technique. PEID sometimes requires conversion to an open technique for optimal management in case of sizeable CSF leaks.

Indications for PELD mainly include lumbar disc herniation (LDH) with symptoms refractory to conservative therapy. In the study by Oster et al. [57], it was already highlighted that surgery in both PELD and open discectomy offers better results than conservative therapy regarding pain reduction, improved functionality, and patient satisfaction. Other indications include lumbar spinal stenosis, recurrent LDH, and certain metastatic lumbar tumors (typically from breast, lung, renal, and prostate cancer), which sometimes require nerve root destruction with radiofrequency ablation. Another indication for PELD is lumbar disc cysts. This rare condition is often refractory to conservative therapy, with a success rate (based on the McNab criteria for cases classified as "good" or "excellent") and PELD of 87.5% according to the study by Ha et al. [58]. However, the literature is still limited on this condition. Another indication is recurrent LDH, previously operated on with an open technique.

Despite its many advantages, which will be discussed later, the PELD clearly has its own complications. Dural tears, nerve root injuries, and recurrence are the three most significant complications that can occur and impact the success of the procedure and the patient's postoperative quality of life. Dural tear is one of the main complications of PELD and is associated with a risk of CSF leaks that can cause postoperative headaches and require dural repair. This risk is particularly relevant in minimally invasive procedures such as PELD, where access is more limited and visibility is reduced compared to an open discectomy. However, advanced visualization techniques and increased surgical experience have helped reduce the incidence of dural tears, although it remains a significant risk to consider. Another complication is the potential for nerve root damage during the procedure. This risk is also present to a greater extent with PELD compared to the open technique, given the limited maneuver space and the proximity of the instrumentation to the nerve root. Damage to the root can lead to persistent pain, loss of sensation, or motor deficits, significantly impacting the patient's quality of life. A correct technique and accurate anatomical identification are essential to minimize this complication.

Our study's third and most exciting complication is hernia recurrence, defined as a recurrence of the same segment within six months of the initial operation. The studies by Guo et al. [59] and Shriver et al. [60] report an incidence of 2-18%, with a higher frequency in male subjects over 50 years of age, smokers with a history of spinal trauma, and those who initially presented with central or paramedian hernias. Among the risk factors for reoperation or reoccurrence, there is also the reduced experience of the surgeon, as indicated in the study by Wang et al. [61], which highlights a significant learning curve with a close correlation between surgical experience and reduction of operating times, postoperative hospital stay, reoperation rate, and VAS (Visual Analog Scale) scores for pain. Finally, operations performed before 2010 showed higher recurrence rates, attributable to the less advanced development of surgical instruments (as indicated by the studies by Yao et al. [62] and Wang et al. [63].

Having spoken about reoccurrence allows us to introduce the discussion about comparing PELD with OLMD (open lumbar microdiscectomy). Contrary to what one might think, several studies, including that of Yoon et al. [64], highlighted similar results regarding the risk of recurrence between the two techniques. However, the study of Kim et al. [65], which involved patients with a five-year follow-up, demonstrates a reduction in the risk of recurrence in cases treated with PELD compared to traditional techniques. Therefore, recurrence of hernia is a frequent complication in both PELD and open discectomy (OLMD), with a frequency of recurrence that, according to some studies, may be similar or even higher in the case of PELD. The main reason it occurs is incomplete disc removal and debris in the spinal canal. The latter induces inflammation and compression of the nerve root and leads to the development of post-discectomy syndrome or failed back surgery syndrome (FBSS), where the patient continues to experience persistent pain even after surgery.

Furthermore, in the presence of residual fragments, nerve compression caused by scarring often requires reoperation. In this case, the risk of dural rupture increases. Therefore, careful management of the patient during the first operation is essential to reduce the likelihood of recurrence and postoperative complications.

Several technical solutions have been explored to counteract the risk of recurrence. One of the first strategies was intradiscal decompression, but it proved to be ineffective and associated with risks of further complications, such as injury to healthy parts of the disc. This technique may reduce disc height and increase the risk of long-term chronic pain, as reported in the study by Yeung et al. [66]. Alternatively, a recent solution proposed in Wu et al. 's [67] study is intradiscal irrigation with a saline solution during PELD surgery. This technique, known as B-PELD (or Bi-needle), involves irrigation of the disc four to six times during surgery with saline solution to remove any residual fragments and reduce postoperative inflammation. Irrigation also promotes nutrition of the disc intervertebral, maintaining a more stable volume and reducing the risk of degeneration-related complications. Compared to the C-PELD technique, which does not require this irrigation, B-PELD has shown promising results. The study by Wu et al., which retrospectively followed 48 patients, showed an improvement in intervertebral disc density (assessed according to the Pfirrmann scale), in the disc/vertebra height ratio, and low back pain scores (VAS), although it did not show statistically significant differences in reoperation rates between the two groups. For the record, applying the Bi-needle technique presents some practical difficulties. For example, using a double needle for intradiscal irrigation can be complex in patients with a narrow transforaminal system. Despite these limitations, B-PELD represents a step toward a more effective and less invasive management of post-PELD recurrences, offering patients a better long-term prognosis.

In addition to the three most common complications already described, PELD may involve some other specific risks, although less frequent: Intervertebral Infections are found in approximately 0.1-0.4% of cases. A percutaneous needle biopsy guided by fluoroscopy can confirm the diagnosis and allow targeted antibiotic therapy if clinically suspected. Only in rare cases, as demonstrated in the study of Yang et al. [68], was an open debridement necessary, with three cases reported in the literature. Postoperative dysesthesia is caused by irritation of the dorsal root ganglion due to the operating instruments and may cause hypoesthesia or dysesthesia in the patient. The floating retraction technique (FRT) developed by Shaw and colleagues, as reported in the study of Cho et al. [69], represents an effective strategy to minimize this complication. Although rare, breakage of the instruments inside the spine is a possible complication. The study by Guan et al. [70] reports only two cases documented in the literature, confirming the low incidence of this event.

Therefore, PELD offers several advantages over traditional open discectomy, starting with less invasiveness. PELD requires an incision of only 0.7 cm, sufficient for inserting a single endoscopic cannula equipped with light, an irrigation system, and a working channel. This approach allows the dissection of anatomical structures, including the spine's joints, muscles, and ligaments. Another significant advantage is the minimal blood loss during the procedure: on average, approximately 5 ± 3 ml of blood is lost, compared to 23 ± 10 ml for a microendoscopic discectomy and 26 ± 15 ml for an open discectomy, as reported in the study by Liu et al. [71]. Furthermore, PELD allows for a rapid hospital stay: the procedure lasts approximately 25-45 minutes, and the patient can usually leave the hospital within 24 hours, as confirmed by the studies by Choi et al. [72] and Sasani et al. [73].

An additional benefit is the possibility of performing the operation under local or epidural anesthesia. This allows immediate feedback from the patient that can help promptly detect any damage to the nerve root. This option reduces the risk of neurological complications compared to general anesthesia, although the latter can still be used if the patient requests it. Finally, PELD's minimally invasive approach reduces damage to the spine's bone elements, decreasing the risk of secondary degenerative pathologies in the long term.

In light of the available evidence, it appears clear that PELD offers numerous benefits, especially when compared to conservative therapy, as demonstrated by the study above by Oster et al. [57]. This surgical intervention is, in fact, more effective than non-invasive treatments alone for pain resolution and improvement of functionality, making it a reasonable choice in many cases. A crucial comparative analysis between PELD and open discectomy (OLMD) comes from the systematic review by Qin et al. [74], which collected and analyzed data from nine studies for 1,585 patients. This study confirms some critical advantages of PELD, such as reduced hospital stays and a quicker return to work for patients. These parameters positively impact patient's lives, demonstrating a real difference compared to open discectomy.

However, the review notes that, in terms of VAS (Visual Analogue Scale for pain hours), ODI (Oswestry Disability Index for disability), duration of the operation, and overall percentage of complications, the two techniques are substantially equivalent. It should be noted, however, that the follow-up of these studies is variable and never exceeds 45 months, which could limit the ability to detect long-term differences. Other studies, such as the aforementioned study by Yao et al. [75], indicate that, over five years, the risk of recurrence tends to be lower in patients treated with PELD, suggesting a potential long-term benefit.

An exciting aspect of PELD is, therefore, its positive effect on patients' quality of life, mainly due to the less invasiveness of the endoscopic approach. With less tissue damage and a quicker recovery, patients report shorter recovery times and a reduction in postoperative complications compared to open discectomy. This result is fundamental for those seeking an adequate but less traumatic solution for the body.

This leads to an important consideration: deciding to opt for PELD or open discectomy should be shared and personalized, considering the clinical aspects and the patient's expectations for quality of life. Although PELD may have higher costs in some countries than open discectomy, its overall improvement in well-being and quality of life often justifies the investment. Open discectomy may have lower costs but at the expense of lower recovery and postoperative comfort benefits. PELD is, therefore, a valid option for patients who wish to minimize the impact of surgery, even if PELD and open surgery show equivalence in strictly clinical parameters. The role of the doctor and the patient remains central as they evaluate and choose the most suitable intervention based on the clinical picture and the patient's specific needs.

It is worth adding that in some percutaneous endoscopic transforaminal discectomy (PETD) procedures, an additional technique called foraminoplasty is used. This technique aims to widen the transforaminal canal by cutting under the superior articular process (SAP) of the vertebra, using instruments such as a side-firing laser or a high-speed burr to ablate the foraminal ligament. However, the study by Li et al. [76] highlights some limitations of the current laminoplasty: the procedure is complex and requires longer intervention times, making it less suitable in highly migrated hernias or stenosis of the lateral recess. Furthermore, using high-speed instruments may increase the risk of postoperative inflammation. In response to these limitations, the study suggests an alternative, faster, and safer technique: cutting the SAP or the hypertrophic osteophyte using specific instruments, such as the trophy or the bone reamer, under fluoroscopic guidance. Although there is no clear evidence of improvement in inflammation yet, this technique represents a more straightforward and equally effective option, offering an exciting starting point for future clinical studies and which we hope will serve as a stimulus to other spinal surgeons to collaborate with biomedical engineers to develop tools that increase the precision of the intervention and its ease.

Minimally Invasive Lateral Lumbar Interbody Fusion for Spinal Degeneration and Deformities

Lateral lumbar interbody fusion (LLIF) is a minimally invasive surgical technique shown in numerous studies to effectively treat bone degeneration and spinal deformity of the lumbar spine. First described by McAfee et al. in 1998 and later altered to Ozgur et al.'s description in 2006, the procedure involves the lateral positioning of a cage in the intervertebral space, allowing the stabilization of the spine and restoration of its alignment in cases of bone deterioration or spinal deformities; LLIF also presents with a similarly high rate of fusion and lower morbidity than traditional methods. It also enhanced postoperative recovery. The technique involves lateral access to the intervertebral space through the psoas muscle and is usually performed under neurophysiologic monitoring to avoid damaging the lumbar plexus. Compared to traditional posterior fusion techniques, it reduces trauma to the posterior musculature and diminishes perioperative morbidity [77].

The patient is initially positioned in a lateral decubitus position, allowing the surgeon to directly approach the lumbar spine's lateral side. A small incision is made, and sequential dilators form a working channel. Intervertebral disc removal is then performed, followed by placing a cage filled with either bone grafts or synthetic substitutes to restore the disc height and achieve fusion [78].The technique is highly versatile and applicable to various spinal pathologies, such as degenerative disc disease, spondylolisthesis, and spinal deformities in adults [79].

There are many advantages to using LLIF for the treatment of the most common causes of chronic pain and disability, particularly degenerative conditions of the lumbar spine, such as herniated discs, spinal stenosis, and spondylolisthesis. Indeed, the process allows restoration of disc height; with a broader cage implanted compared to posterior techniques, LLIF can significantly restore the intervertebral height, and therefore, indirectly, neural compression and the symptoms associated with stenosis are improved considerably [77]. The large surface area of the cage implanted in LLIF promotes osseous integration. When applied to degenerative diseases, the fusion rates using this technique were reported in the literature to be more than 90% [78]. Moreover, the less invasive nature of LLIF results in less blood loss, shorter operating times, and shorter hospitalizations compared to standard open posterolateral interbody fusion (PLIF) [77].

LLIF is especially useful in treating spinal deformities, such as adult degenerative scoliosis and kyphoscoliosis, since it allows for significant coronal and sagittal plane corrections. In this regard, LLIF provides good coronal deformity correction with lateral access to the spine and placement of interbody spacers that restore alignment. Abe et al. demonstrated that a combined anterior-posterior approach, including LLIF, was associated with significant rotational deformity correction in patients with kyphoscoliosis [80]. More importantly, LLIF fills the need to restore lumbar lordosis in sagittal imbalance. Large interbody cages encourage the restoration of segmental lordosis and decrease the need for additional posterior osteotomies [79]. In more severe deformities, LLIF has been commonly performed in combination with posterior instrumentation in a staged manner, improving correction while minimizing surgical complications [80]. Nevertheless, spinal deformities remain a significant challenge to treat because of their biomechanical complexity and associated comorbidities.

Comparison studies support the advantages of LLIF over traditional techniques. Miscusi et al. compared LLIF to OLIF, and both procedures were equally effective in treating degenerative disc disease, with clinical results and fusion rates improving similarly [81]. LLIF and OLIF have also been shown to have fusion rates of over 90%, supporting the reliability of the lateral surgical approaches [81]. LLIF was associated with slightly increased rates of transient lumbar plexus irritation from the transpsoas approach, but OLIF eliminated this complication by employing an oblique corridor [27]. The procedure's advantages include its favorable recovery profiles, which include shorter hospitalizations, early return to activity, and lower complication rates. Since LLIF is an MIS, it has favorable recovery profiles. Most patients undergoing LLIF require less hospitalization than patients undergoing PLIF or TLIF, with an average hospitalization of two to four days [77]. The pain and function scores dramatically improved in the first three months following the surgery. The operation offers long-term symptom alleviation, with most patients experiencing baseline function within six months [79].

Longitudinal studies have shown that LLIF maintains its clinical and radiological benefits over time and thus is also a stable solution in the long run [77]. Despite its many advantages, LLIF has some limitations: The transpsoas approach can irritate the lumbar plexus, which may manifest as transient thigh pain, weakness, or numbness. Neurophysiologic monitoring is essential to minimize these complications [78]. LLIF is less suitable for treating the L5-S1 segment due to iliac crest obstruction and requires alternative approaches [77]. Surgeons face a steep learning curve to adequately perform LLIF, a challenging technique with unique instrumentation [79]. However, technological and technical innovations continue to expand the capabilities of LLIF. In particular, introducing robotic systems and intraoperative navigation significantly increases the accuracy and safety of LLIF procedures [79]. Furthermore, the prone transpsoas LLIF has been developed by combining the advantages of the lateral approach with the flexibility provided by the prone positioning and therefore the ability to expand the deformity correction while reducing the operative time [79]. Furthermore, growth factors and bone substitutes are being studied to improve the outcome of the fusion in the most challenging cases [77]. In conclusion, the minimally invasive LLIF has allowed remarkable progress in treating degenerative diseases and spinal deformities. The combination of effective biomechanical correction, minimal invasiveness, and an accelerated recovery period makes the LLIF one of the most preferable alternatives for many patients. Proper patient selection remains also essential for long-term success, for which studies remain limited.

Percutaneous Vertebroplasty and Kyphoplasty for Osteoporotic Vertebral Fractures 

Vertebral fractures caused by osteoporosis are one of the most frequent and debilitating complications of osteopenia. They compromise the patient's quality of life and significantly increase the risk of disability and mortality. In recent decades, percutaneous vertebroplasty (PVP) and percutaneous kyphoplasty (PKP) have been used as minimally invasive options for the treatment of these lesions, offering promising clinical results in terms of pain reduction, restoration of function, and reduction of hospital stay. Osteoporosis is a systemic disease characterized by reduced bone mineral density and alterations in bone microarchitecture. These alterations increase the risk of fractures, especially at the vertebral level, causing acute pain, spinal deformity, and functional limitation. If left untreated, these fractures can evolve into chronic pain syndromes and further worsen the quality of life [82,83]. Traditional treatment options (conservative therapy with analgesics, bed rest, and spinal support devices) do not always guarantee optimal recovery. For this reason, PVP and PKP have significantly improved the therapeutic prospects for this type of patient. 

PVP was introduced as a minimally invasive procedure in the 1980s. It involves injecting bone cement (usually polymethyl methacrylate, PMMA) into the fractured vertebral body via a transpedicular or parapedicular approach. The technique provides immediate mechanical support and stabilizes the fracture, relieving pain caused by micrometric movement of the bone fragments [84]. The main indications for PVP include painful osteoporotic vertebral fractures not responding to conservative treatment, Spinal instability diagnosed by imaging and Vertebral neoplasms with a lytic component, such as multiple myeloma or bone metastases. 

Numerous studies have documented significant improvement in postoperative pain in patients undergoing PVP. Filippiadis et al. demonstrated that PVP relieves pain in over 90% of cases, substantially improving short-term quality of life [85]. In addition, PVP is associated with a relatively low risk of complications, making it the first choice for elderly or frail patients. Later, PKP developed, representing an evolution of PVP. It involves introducing a balloon into the vertebral body, which is inflated to create a space and restore vertebral height before cement injection. This step allows partial correction of fracture-associated kyphosis, improving spinal alignment [84]. The main advantages of PKP include more marked pain reduction than PVP, Improved mechanical function due to restoration of vertebral height, and lower risk of cement leakage due to creating a more defined space. 

A study by Xiang et al. compared the efficacy of PKP and PVP in patients with vertebral fractures associated with multiple myeloma. The PKP group significantly reduced pain, and the patients were more satisfied [85]. A comparative analysis of the two procedures reveals that both are highly effective in controlling pain and improving quality of life, but with some substantial differences including pain and function: both techniques are effective, but PKP offers a slightly more significant advantage in functional restoration. In terms of risks and complications, PVP is associated with a higher risk of cement leakage, which can cause pulmonary embolisms or compression of nerve structures. PKP, owing to the balloon, significantly reduces this risk [86] and restoration of vertebral height; hence, PKP is more effective in correcting kyphotic deformity [87]. 

One of the main advantages of PVP and PKP is their minimally invasive nature, which leads to faster recovery times and fewer days of hospitalization. Patients can usually return to daily activities within a few days of surgery, significantly improving their psychological and social status [83]. Furthermore, these procedures reduce the need for long-term analgesic drugs, helping to decrease the risk of opioid dependence. Lamy et al. highlighted that minimally invasive treatment of osteoporotic fractures avoids the complications associated with prolonged conservative therapy, such as progressive loss of density and secondary disability [86]. Despite their benefits, PVP and PKP are also controversial. Some studies have questioned the long-term efficacy of these procedures, suggesting that pain improvements may be attributable to placebo effects rather than proper biomechanical stabilization [85]. Furthermore, the possibility of new fractures in adjacent segments represents a non-negligible complication, potentially related to changes in spinal biomechanics after surgery. Another area of debate concerns the cost of the procedures, with PKP being significantly more expensive than PVP due to the materials used. Therefore, the choice of procedure should be individualized, considering the patient's clinical condition, available resources, and individual preferences [87]. 

PVP and PKP techniques continue to evolve, with the development of new bone cement materials and devices designed to improve the procedure's accuracy and safety. In addition, advanced imaging, such as intraoperative computed tomography, enhances the ability to plan and monitor in real-time during procedures [83]. Future studies should focus on optimizing clinical protocols and identifying patient subgroups that benefit the most from each technique. In addition, it is necessary to continue evaluating the long-term effects of these procedures, considering both the benefits and potential risks [84]. 

To summarize, PVP and kyphoplasty represent two highly effective and safe therapeutic options for treating osteoporotic vertebral fractures. Due to their minimally invasive nature, these techniques allow for rapid pain relief, improved function, and reduced hospital stay, significantly benefiting patients with osteoporosis. However, the choice between the two procedures must be carefully evaluated, considering the specific needs of the patient and the available resources. With continued technological advances and increasing clinical experience, these techniques will continue to play a crucial role in managing osteoporotic vertebral fractures.

MRI-Guided Cryoablation for Metastatic Spine Disease

Over the past few years, image-guided cryoablation has become increasingly crucial for the surgical treatment of skeletal metastatic illness, and several benefits have been identified. For spinal disorders, minimally invasive percutaneous image-guided procedures are both safe and successful. Percutaneous image-guided cryoablation allows for improved imaging of the surgical peripheries compared to heat ablation techniques. Monitoring the ice ball with intermittent CT or MRI is an obvious advantage of cryoablation. Neurological complications can be minimized by pre-procedural planning and intra-procedural ablation zone management [88].

PCA is a technique that has been recently adopted in minimally invasive neurosurgeries. This technique involves the placement of a needle applicator and a cryoprobe into a spinal tumor using imaging guidance. Most commonly, Argon gas at room temperature is used to cool the tip of the cryoprobe to −40 °C rapidly, allowing the formation of an ice ball, which is readily identified by imaging techniques such as CT [89]. The monitoring of iceball through imaging techniques such as US, CT, and MRI guidance has an advantage over thermal ablation as it allows real-time follow-up [90].

Moses et al. conducted a prospective trial for cryoablation for head, neck, and spine malignancies. To obtain access to the spinal canal and real-time intraoperative imaging during cryoablation, a total of 14 patients with metastatic spine illness were examined and tracked using CT and MRI. The mean preoperative Karnofsky Performance Status score was 79.3 (range 35-90), and the average age was 54.5 years. There was an average clinical follow-up of 9.8 months (seven to 943 days) and an average radiographic follow-up of 7.1 months (25-772 days). Seven out of 10 patients with epidural illness who had postprocedural imaging showed steady or decreased radiographic disease load (5/7). Meanwhile, 63% (5/8) of the patients who underwent bone ablation during treatment and had postoperative imaging showed evidence of bone regeneration. Values on the VAS were obtained before and after surgery, and these values significantly dropped after ablation. No postoperative complications have been noted in any patients [91].

In a study, 39 patients between 2011 and 2017 were observed for post-procedural MRI to detect any remnants of spinal osseous metastases after cryoablation [92]. This was deduced efficiently by analytically comparing the post-cryoablation MRI. In a meta-analysis by Yang et al., different screening techniques such as FDG PET, CT, MRI, and bone scintigraphy (BS) were compared statistically for the diagnosis of bone metastases. The sensitivity of the MRI was 96.0%. It was concluded that MRI and PT define the margins of the surgical site with higher resolution and more accurately remove the remains of the tumor than CT and BS [93,94].

The safety of ablating tumors was studied by Tuncali et al. The study aimed to demonstrate cryoablation's safety using the MRI system setup. Two presacral tumors enclosing the sacral plexus underwent ablation connected to sacral plexus branch ablation, whereas five tumors were treated without causing damage to the operative site. MRI was effective in detecting and tracking iceball deposition in every instance. The tumor was easily distinguished from the healthy tissue thanks to the intervention and integration of MRI, which also helped with targeting. The ice ball was readily identified and marginalized using standard 0.5-T MRI sequences. This allows the surgeon autonomy over the cryoablation area and enables them to modify the ice ball's placement as necessary. MRI is utilized to prevent nearby vital structures from freezing [95].

In summary, MRI-guided cryoablation for metastatic bone diseases is highly sensitive and can remove the entire tumor without leaving any remnants. Although CT- and sonography-guided radiofrequency ablation are used to control many lesions, MRI has clinically proven more precision and accuracy while allowing the surgeon to control the surgical parameters.

Robot-Assisted Minimally Invasive Techniques for Spinal Tumor Resection 

Over the past few decades, advances in robotic technologies have revolutionized spinal surgery, resulting in improved precision and less damage to surrounding healthy tissues. This has encouraged the acceptance of minimally invasive techniques for the resection of spinal tumors, which requires the highest precision, preventing complications and preserving vital surrounding structures. The current article will review the progress made in robot-assisted spinal surgery, with particular attention to tumor resection. 

Minimally invasive spinal surgery (MIS) was developed to decrease the extent of surgical trauma and postoperative pain and to speed up recovery times. Large incisions are often required in traditional open surgical techniques, which can also potentially compromise spinal stability and place patients at increased risk of infections and other complications [96]. One of the main problems related to minimally invasive techniques is achieving sufficient accuracy when working in confined spaces with limited visibility. 

Robotic technology is considered a feasible solution to overcome these difficulties. Robotic systems offer unique advantages, including improved intraoperative visualization and the ability to plan predefined surgical paths with millimeter precision. In particular, robotic systems used in spinal surgery, such as Mazor X or ROSA Spine, are designed to integrate advanced imaging and navigation systems [97]. The use of robotics in spinal surgery offers a higher level of accuracy, which becomes even more advantageous in complex cases such as tumor resection. An essential component of this procedure is intraoperative navigation systems based on three-dimensional images produced by computed tomography or fluoroscopy. These images are incorporated into the robotic system software, thus allowing the surgeon to define safe surgical trajectories [98]. Concerning the advantages, robotic-assisted surgery provides better localization and removal of tumors with greater accuracy and less damage to surrounding tissue [99]. MIS reduces postoperative pain and results in shorter recovery times compared to conventional techniques. Robotic systems offer greater control in the operating room, which means less room for human error. The introduction of robotic systems allows for the standardization of surgical protocols, which provides consistent quality of surgical outcome measures [97]. 

Despite significant progress, robotic surgery still needs to be improved due to several factors, including high equipment costs and the need for specialized surgical training. The learning curve can also be significant, requiring time and resources to familiarize oneself with the technology [97,99]. The removal of spinal tumors surgically is one of the most challenging tasks in neurosurgery. These tumors, whether intradural, extradural, or metastatic, need operative treatment to preserve neurological function and keep the spine stable. Preoperative planning is a critical factor in the successful performance of robotic surgery. Modern robotic platforms allow for virtual simulation of the surgery before the actual procedure, enabling the assessment of pedicle screw placement and determination of access for tumors [98]. One example is the Mazor X system, which is acknowledged for planning screw insertion with high accuracy, therefore minimally necessitating correction due to its execution [100]. During the operation, the robot guides the surgeon to stay on the predetermined track. This method primarily benefits tumor resection in an anatomically poorly accessible region. Robotic navigation offers high accuracy of the instruments' placement and avoids vital structures like the spinal cord and spinal nerves [99]. A comparison of robot-assisted surgical interventions with traditional surgical methods revealed that robotic systems increase the precision of implant positioning while significantly reducing the length of operations [100]. Moreover, the rate of intraoperative complications is lower in situations operated using robotic technology. There is a lot of clinical evidence supporting the efficacy of robotics in resecting spinal tumors. For example, a retrospective analysis by de la Torre et al. showed that those with robot-assisted surgery had fewer postoperative complications and shorter hospital stays than those with the traditional technique [99]. In the same spirit, Zhang et al. underscored how robotic navigation reduced the risk of error during pedicle screw insertion for improved results in the long run [98].

Advances in robotics are giving rise to new technologies that will likely continue changing the face of spinal surgery. These include the following: 1) Integration of AI: AI in robotic systems can support improved accuracy in surgery, where robots dynamically adjust to the patient's condition. 2) Portable robotics: More compact and portable systems could make robotic surgery accessible even in smaller hospitals. 3) Remote surgery: Telesurgery, enabled by robotic systems, could allow surgeons to operate remotely, improving access to care in remote locations. 

The minimally invasive robot-assisted technologies in the final analysis represent a revolution in the history of spinal surgery, with improved results on accuracy, safety, and recovery time. While some challenges remain, such as the financial impacts and specialized training required, the future is bright for robotics in neurosurgery. Further innovation can only lead to standardized practices and improved clinical outcomes.

Robotic-Assisted Pedicle Screw Placement 

Pedicle screw placement is a technique used in spinal surgery to stabilize and fuse the spine vertebra for a wide range of diseases like unstable vertebral injuries, osteodegenerative disease, or scoliosis. During this surgical intervention, accuracy in screw placement is of prime importance to avoid injury to adjacent structures, now that possible complications can be extremely severe and include nerve root injury, spinal cord injury, vascular injury (especially to the azygos vein, intercostal arteries, inferior vena cava, and thoracic aorta), CSF leak, and damage to visceral structures like the esophagus, pleura or lungs [101]. Although it is considered a relatively safe surgery where complications rarely arise, robotic systems can significantly help reduce the risk of human error by improving accuracy through aids in structure registration, depth perception, and visual and tactile synchrony. The robotic component comprises an optical tracking device, a surgical controlling workstation, and the robotic arm. In essence, the optical tracking device locates the spatial positions of the robotic arm in real time. At the same time, the controlling workstation processes the images, aids with surgical planning, and calculates coordinates to control the robotic arm.

When comparing a robotic-assisted pedicle screw placement to a freehand screw placement, it is essential to have a standard parameter of measurement to evaluate surgical outcomes. The Gertzbein-Robbins classification grade sets an international parameter that allows us to compare both techniques, where a “perfect pedicle screw placement” or grade A is defined as a screw entirely within the pedicle and a “clinically acceptable pedicle screw insertion” or grade A+B is categorized as a screw placement where ≤3 mm sits outside the pedicle, without any relevant clinical complications [102].

When compared in a meta-analysis, the results demonstrated that patients undergoing a robotically assisted pedicle screw placement had a 1.68-folds higher likelihood of achieving a perfect pedicle screw insertion and a 1.54-folds higher probability of achieving a clinically acceptable pedicle screw insertion about the freehand technique [103]. Moreover, complications after pedicle screw placement, including hardware failure, wound infections, neurological deficits, and surgical revision, accounted for 6.91% in the robot-assisted group and 20.6% in the freehand group [104,105]. Skin-to-skin operative times favored the freehand technique groups, with a mean difference of 22.7 minutes (95% CI: 6.57-38.83, p = 0.006). However, it can be attributed to more preparation work for the robot system and to the learning curve linked with robot-assisted surgery [106,107].

Robotic surgery tends to be an attractive topic of interest for the scientific community nowadays due to its direct impact on minimizing surgical errors by aiding in performing complex procedures with the highest level of precision. This cutting-edge technology aids tremendously worldwide and opens doors to procedures that were once considered impossible. However, the robotic operational costs with extended surgical times are variables that should be considered, especially in surgeries with relatively low complication rates.

Smart Glasses for Fluoroscopically Guided Spinal Surgery 

In most spinal surgeries, surgeons are forced to turn their heads away from the surgical field to visualize a series of intraoperative support monitors, which can lead to potential discomfort, technical difficulties, and medical errors. Smart glasses are wearable visual aids that provide a second screen in front of the surgeon’s eyes without blocking the surgical field of view, allowing them to stay focused while having an assisted real-time augmented and mixed-reality visualization during the surgery. With these glasses, surgeons can navigate and plan through the surgery more efficiently by displaying guidance, real-time imaging, and patient information [108]. These glasses are closer than fluoroscopic monitors in the operating room, allowing for a precise view and reducing possible radiation exposure during procedures like pedicle screw placements. Most importantly, during these delicate, minimally invasive procedures, intraoperative support devices must be implemented to aid surgeons and enhance their vision in narrow and limited surgical fields [109].

When comparing fluoroscopically guided spinal surgery with and without smart glasses, the radiation exposure time and insertion time were significantly shorter than the group without smart glasses (i.e., 11.6 s vs. 15.0 s, p = 0.000001; and 14.5 s vs. 19.3 s, p < 0.00001, respectively) [110]. Moreover, the number of “surgeon head turns” to view the fluoroscopic monitor in the smart glasses group was 0.10 +/- 0.31 times (range 0-1 times), compared to 82.4 +/- 32.5 times in the group without smart glasses. Operative times (mins) were also shorter in the group with smart glasses (100.2 +/-10.4min vs. 105.5 +/- 14.6min), which relates to a shorter average operational cost during surgeries and a higher efficacy. Postoperative complications were insignificant between the two groups. However, blood loss (mL) was also lower in surgeons operating with smart glasses when compared to those without (55.1+/-35.1 mL vs 66.3+/-38.3 mL) [111,112]. Implementing smart glasses in spinal procedures ensures a more comfortable environment in the operating room, where the surgeon can perform procedures with full-body mobility. The most significant impact of smart glasses is the shortening of the average operating time, with a reduced intraoperative radiation exposure time. Less head turning during complex procedures also ensures more concentration during the surgery and fewer possible complications.

Real-Time Ultrasonography-MRI Fusion Navigation in Spinal Surgery 

The use of intraoperative US has seen a trend in spinal surgery applications. Recent research has been conducted to understand the use of iUS. Navigating with iUS integration has been marked as safe, economical, and effective. The most common approach of iUS is seen in pedicle screw instrumentation. Anatomical margins are well visualized by iUS modalities such as standard B-mode, Doppler, and contrast-enhanced ultrasonography. It eliminated the radiation side effects and enhanced the efficiency of the surgery by targeting the correct neurovascular structure in various spinal surgeries. A study by Madhav R et al. examined how technology has advanced spinal surgeries and how numerous intraoperative imaging procedures, including CT, MRI, and US, are frequently carried out. It was demonstrated that iUS showed superior neurovascular identification, recognizing landmarks and bone structures around the organ. iUS displays PSI and registration correctness. It is frequently employed in spine operations and procedures such as fusions, decompressions, and fracture reductions because of its capacity to discriminate between margins. It lessens the financial strain and radiation exposure [88]. Liu et al. published a case report. 

While C-arm fluoroscopy, a commonly used fluoroscopic guidance technique, may not be appropriate for every patient, it is frequently used in clinical settings. Standard settings may be challenging to utilize due to several issues, and fusion imaging, a radiation-free technique that combines real-time US with magnetic resonance imaging (MRI), is becoming more popular. An intraspinal lesion at the L1 level was identified using volume navigation technology (VNT)-based fusion imaging of the US and MRI. The intraspinal lesion was located, and surgery was recommended for a 37-year-old pregnant woman in her 18th week of pregnancy. A minimally invasive procedure significantly reduced her symptoms, and at the 40th week of pregnancy, a healthy baby was born [113]. This revealed a selective preference for the imaging technique in patients who cannot be exposed to radiation. Thus, iUS is more favorable in exceptional cases, and a shift could be seen toward it to reduce potential radiation effects. It relieves the pain in skeletal metastases and is palliative toward spinal decompressions. It has probable local tumor control and primary efficacy. It is a promising method for palliation and remodeling bone metastases such as spine [88,114]. It accurately locates the tumor and helps in complete removal without damaging the surgical site. The delivery and tracking of thermal injury in the ablation of tumors improves accuracy and efficiency. The contrasting real-time visuals give surgeons better control over the surgery. The changes at the surgical site can be detected immediately, preventing surgical injuries and complications. The innovations have provided better control over the surgery.

Innovations and emerging technologies

AI-Assisted Minimally Invasive Tumor Ablations

Minimally invasive tumor ablation has transformed cancer treatment, providing patients with a method for tumor eradication that entails minimal recovery time and diminished risk relative to conventional surgery. A notable progression in this domain is the amalgamation of AI with ultrasound-guided focused ultrasound (USgFUS), a noninvasive method employing high-intensity focused ultrasound (HIFU) to target and obliterate tumors precisely. HIFU concentrates high-energy US waves on targeted tissues, producing thermal effects that ablate tumors. This technology has become prominent as a noninvasive approach to cancer treatment, substantially decreasing the necessity for incisions and lowering the risk of complications. Nonetheless, obstacles persist in the precise monitoring and direction of treatment. Conventional B-mode US imaging, dependent on grayscale variations to depict tissue ablation, encounters constraints in accurately defining ablation margins and tracking real-time temperature fluctuations during treatment. This imprecision may result in inadequate tumor ablation, requiring multiple interventions and endangering adjacent healthy tissue. 

To get around these problems, some authors suggest using an AI-driven framework for US-guided focused US. This framework would include an AI segmentation model based on the Swin-Unet architecture. This model combines the best features of the Swin Transformers and U-Net architectures to make multi-scale feature extraction easier. This makes it very good at finding small changes in tissue that mean a tumor has been removed. In experiments outside of living things using chicken breast tissue, the AI-assisted USgFUS method worked amazingly well, achieving 93% segmentation accuracy. It was proven even more by comparing images of areas that had been ablated before and after the procedure. There was a strong link between the two, which shows that the model could be used for accurate, real-time monitoring in clinical settings [115]. 

When AI is added to the USgFUS method, it could significantly improve the accuracy of tumor ablation in clinical settings. The AI model automates the segmentation process, which makes the results more consistent and less reliant on operator skill. This level of accuracy is needed to remove cancerous tissue altogether while causing as minor damage as possible to healthy tissue. This is especially important in complicated cases where tumors are close to vital structures. Real-time AI-assisted monitoring could also be used in clinical workflows to help plan treatments and make surgery decisions, making patients safer and more effective. Doctors can improve the treatment process by changing the sonication parameters on the fly when they can see the tumor ablation happening in real-time. In addition, AI could help determine the best way to treat the whole tumor while minimizing damage to nearby tissues, making the treatment plan more personalized and accurate [116]. Knowing that doctors can watch tumors being removed in real time lets them change the sonication's parameters on the fly, which ultimately improves the treatment. 

AI might also help figure out the best way to cover the whole tumor while minimizing damage to the nearby tissues. This would lead to a more personalized and accurate treatment plan [116]. Even though the study's results were positive, many problems still need to be fixed before this AI-assisted method can be used in clinical settings. On the other hand, the tests were done on ex vivo tissue, which is less complex than human tissue still alive. More research needs to be done to see if the model works in living tissues, especially with a wide range of tumor types and organ locations. The model depends on hyperechogenicity, an essential property of US imaging. This means that it might not work well in tissues where this phenomenon is less noticeable. The training dataset must include a broader range of tissue types and sonication parameters to make the model more useful in clinical settings. In addition, AI models could help make treatments more accurate, but using them in real life would mean solving problems by standardizing data and ensuring the models are correct. AI models need to be put through many tests to ensure they are reliable and can be used repeatedly with different types of patients and in various clinical settings. Researchers, clinicians, and regulatory agencies must collaborate to develop standard protocols for using AI to help with tumor ablation [116]. US-guided focused US and AI are a big step forward in minimally invasive tumor ablation. AI could make tumor ablation safer and more effective by improving monitoring in real-time, making the procedure more accurate, and automating tasks like segmentation. Moving forward, this will make it possible for noninvasive treatments to work just as well as traditional surgery. As AI technology keeps improving, its uses in oncology are expected to grow. This will give cancer patients new hope and move the field of precision medicine forward [117].

Robotics-Assisted Surgeries for the Brain and Spine 

Robotics integrated into neurosurgery aims to improve patient outcomes of traditional manual methods by improving the accuracy, consistency, and efficiency of many surgical procedures. Robotic systems are utilized in a diverse range of cranial and spinal applications, including the precise positioning of implants, careful tumor resections, and improved protection of critical neurovascular structures. Despite its advantages in minimally invasive procedures, robotic surgery often requires longer operative times due to the setup of robotic instruments [118]; it also involves training methods such as virtual reality (VR) simulations and cadaver-based practice for surgeons to have the necessary skills to utilize these systems effectively. Innovations in VR, particularly patient-specific simulations, and advancements in robotic design are helping bridge the gap between technology and clinical application [119]. In addition, robotic systems taking part in neuroendoscopy offer further benefits, such as reducing surgeon fatigue by serving as dynamic and stable endoscope holders [120]. Another important role of robotics in neurosurgery is in stereotactic surgeries. Robotic-assisted techniques significantly enhance the accuracy and safety of brain tumor management, particularly in biopsies. 

Robotic systems integrated with stereotactic navigation offer precise, minimally invasive pathways to obtain tissue samples. It allows neurosurgeons to merge preoperative imaging with advanced software, generating a detailed surgical trajectory. The robotic arm then follows this plan with high precision, enabling smaller incisions and reducing the risk of complications. The benefits of robotic-assisted techniques lie in diagnostic accuracy and minimized patient recovery time; they offer a safe and effective method for obtaining diagnostic tissue samples, particularly in challenging anatomical locations [121]. Robotic-assisted spine surgery offers significant advantages over freehand techniques, including fewer complications and improved outcomes. These benefits stem from enhanced surgical precision and instrumentation accuracy and require detailed preoperative 3D planning. Enabling surgeons to modify the procedures based on each patient's anatomy. In robotic-assisted spine surgery, the MAZOR is well known for these procedures. It also minimizes intraoperative radiation exposure by 80% compared to freehand techniques and represents a significant advancement in minimally invasive spine surgery [122]. When treating spinal tumors, robotic-assisted surgery is used for pedicle screw placement, biopsies, and vertebral augmentations like vertebroplasty [123]. Among spinal surgeries, the pedicle screw fixation is one of the most performed surgeries; robotic-assisted surgery has arisen as a less invasive alternative, it reduces postoperative complications, but it requires, as mentioned before, extensive preoperative planning, taking into consideration that injury of sensory and motor nerves as a complication, robotic-assisted spinal surgeries have shown better pedicle screw placement precision and reduced radiation exposure [124].

VR and AR are relatively new technological tools that have recently been useful in preoperative neurosurgical planning, surgical training, and navigation. VR involves a user interface that places the user in a virtual system that impedes the natural world while creating a virtual artificial world the user can interact with. In this case, the virtual world can be designed as a virtual environment (non-immersive) or a realistic substitute for the real world (immersive) [125]. AR, conversely, differs from VR in that there is a fusion of the natural and virtual worlds. In this model, superimpositions (holograms) can be placed over the real-world environment, allowing for a simultaneous perception and interaction of both the natural and virtual worlds. Both models use stereoscopic glasses for visualization and controllers that will enable the interaction and feedback of the virtual world [126]. Neuronavigation, mainly, is an area in which VR and AR excel. A 3D model of an actual patient's head can be created and interacted with (especially for surgical planning) by fusing a real-time MRI and constructing a virtual model. This enhances the immersive experience of intraoperative navigation now that essential landmarks and critical neurovascular structures can be highlighted beforehand and analyzed on-site [127]. 

The same principle can be applied to microscopic neurosurgery, where preoperative 3D images are superimposed on the microscope's eyepieces, enhancing navigation and spatial awareness. AR and VR have also been reported to be used as diagnostic tools for neuroimaging techniques. Its clinical application can augment the diagnostic efficacy of cerebrovascular and neuro-interventional surgery [128]. Neurointerventionalists can use this technology to develop a VR immersive model based on real-time, patient-accurate anatomy for better planification, training, and procedural skills [129]. Complex neuro-interventions and procedures requiring a combination of interventional and surgical methods can also benefit from using VR models to evaluate and plan the intervention. Education and training benefit the most from AR and VR applications now that they can simulate multiple accurate procedures with anatomically correct structures as often as needed. This virtual environment can be used in teaching hospitals to quantify surgeon performance, assess surgeon proficiency, and track individual progression among students.

Patient selection and clinical outcome optimization

Criteria for Minimally Invasive Techniques 

Age is one of the most important predictors of postoperative outcomes. Aging often alters various biological systems and physiological responses, which decides the trajectory and plan of post-surgical rehabilitation. Elderly patients are usually multi-morbid, predisposing them toward a higher surgical risk. Minimally invasive procedures are beneficial in such patient groups. In older patients, the duration of surgery is pivotal. Traditional open surgeries involve a higher risk of tissue damage, longer recovery time, and increased risk of post-surgical complications. A study by Yolcu et al. proved that MIS is a safer option for the elderly with spinal canal stenosis due to shorter surgical hours, lower risk of spinal fluid leaks, fewer post-surgical complications, and earlier hospital discharge time, with many treated in outpatient clinics [130]. Tumor location and size are two other important factors. Minimally invasive procedures such as endoscopic keyhole surgeries have made unapproachable tumors easily accessible, especially in the posterior fossa, skull base, and spinal column. For instance, giant pituitary adenomas approached with an endonasal endoscope result in a lower recurrence rate with a significant decrease in patient mortality and morbidity, as mentioned in Rahimli et al [131]. 

**Figure 1:**
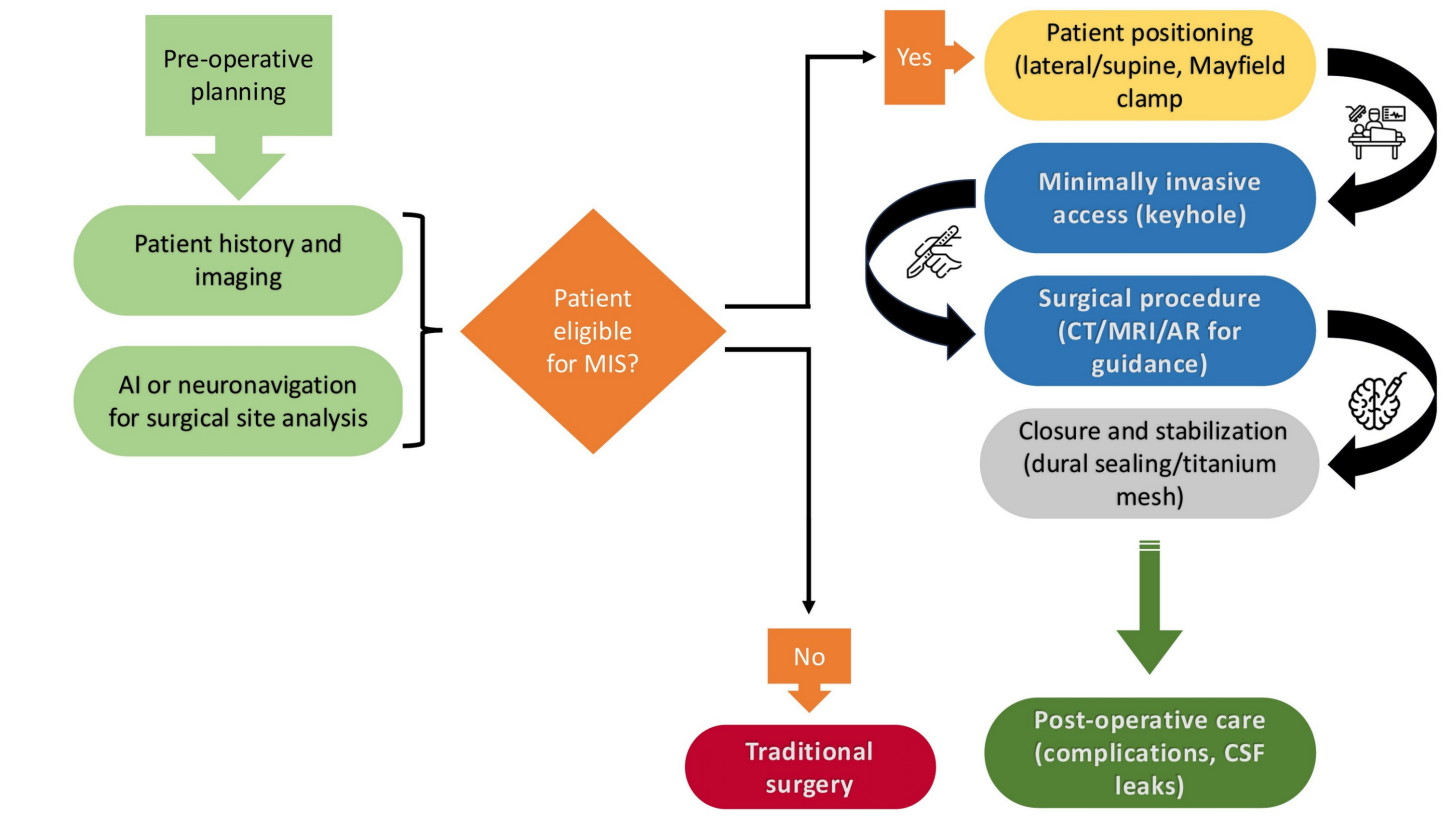
Minimally invasive surgery workflow The figure demonstrates a brief insight into the order of decisions made in minimally invasive surgery. Original image created by the authors.

Patient frailty is another essential factor to consider when exploring surgical options. Patients with high frailty scores benefit the most from minimally invasive surgical techniques. In a study by Passias et al., frail patients treated with circumferential MIS had a significant reduction in blood loss, postoperative pain, recovery time, and hospital stay compared to patients who had undergone traditional open surgery. This method is highly beneficial for patients who may struggle to meet the physical demands of open surgery due to altered heart function and limited lung capacity, topped with lower resilience for recovery [132]. Patient history has a significant impact on surgical outcomes. Postoperative complications are higher in patients with cardiovascular and respiratory diseases. MINPs are a safe bet for patients with comorbidities as they reduce the likelihood of adverse clinical outcomes. In the past decades, MINPs have gained considerable interest among Neurosurgeons. Proper patient selection is a complex process that requires careful assessment. Age, tumor location, and overall health are key in determining an appropriate surgical candidate. The benefits of minimally invasive procedures, such as decreased postoperative recovery time and reduced complication risk, are seen in old and frail patients. In addition, tumor location and technical feasibility determine if minimally invasive or traditional techniques are more favorable. Figure 1 presents a better understanding of the process:

Surgical and Non-surgical Interventions for Lumbar Spinal Stenosis

Surgical and non-surgical approaches to treating lumbar spinal stenosis aim to alleviate symptoms and improve quality of life, though their relative effectiveness remains uncertain [133]. Lumbar spinal stenosis is characterized by the narrowing of the spinal canal in the lumbar region, leading to pain, mobility issues, and functional impairment. This condition significantly impacts daily life, especially among older adults. Low-quality evidence suggests no significant differences in pain-related disability between surgical decompression (with or without fusion) and conservative multimodal care at six months and one year. However, decompression showed a slight benefit at 24 months. Similarly, minimally invasive decompression and epidural steroid injections demonstrated mixed short-term results, with no clear long-term advantages favoring either approach. Interspinous spacer implantation showed improved symptom severity and physical function over conservative care in the first year, though the evidence remains limited. Surgical interventions are associated with complication rates ranging from 10% to 24%, including issues such as fractures, respiratory distress, and the need for reoperation. In contrast, no adverse effects have been reported for conservative treatments, making them safer alternatives, particularly for patients with elevated surgical risks. Despite these findings, the quality of evidence is generally low, with significant variability in study design, treatment protocols, and outcome measures. The absence of clear benefits for surgery over non-surgical treatment underscores the importance of carefully informing patients about the risks and limitations of each approach. Future high-quality research is essential to establish standardized protocols and clarify the relative advantages and disadvantages of surgical and conservative treatments for lumbar spinal stenosis.

Surgical Outcomes in Minimally Invasive Spinal Cord Injury Procedures 

Minimally invasive surgical approaches for spinal cord injury focus on reducing procedural trauma and improving outcomes through precision and adaptability. Chronic pain management remains a key aspect of treatment, with pain types often categorized as neuropathic, nociceptive, or mixed [134]. Neuropathic pain is particularly persistent and challenging to address, necessitating a combination of pharmacological, non-pharmacological, and surgical strategies. Among pharmacological options, gabapentinoids such as gabapentin and pregabalin have shown effectiveness, with pregabalin offering additional benefits in alleviating anxiety and sleep disturbances. Alternative drugs like mirogabalin are emerging as promising candidates, while traditional options like lamotrigine and valproate demonstrate limited analgesic effects. Minimally invasive methods, including botulinum toxin injections, lidocaine infusions, and intrathecal therapies, have shown potential in reducing pain intensity. Complementary interventions like transcutaneous electrical nerve stimulation (TENS), physiotherapy, and advanced neurostimulation techniques (e.g., transcranial direct current stimulation and repetitive transcranial magnetic stimulation) offer additional avenues for pain relief. However, variability in outcomes and methodological limitations underscore the need for rigorous and larger-scale studies. Preclinical advancements are central to improving the translational pathway for spinal cord injury treatments [135]. Novel experimental models, such as those employing kyphoplasty balloons or controlled contusion techniques, enhance the consistency and reproducibility of research findings. These models allow for precise modulation of injury severity, enabling a more robust evaluation of therapeutic approaches. Rigorous methodological design and independent replication of promising results are critical to bridging the gap between preclinical findings and clinical applications. The integration of minimally invasive surgical techniques, innovative pain management strategies, and refined research models holds significant potential for advancing the treatment of spinal cord injuries. A comprehensive and multidisciplinary approach, grounded in rigorous research and patient-centered care, is essential for optimizing outcomes and addressing the diverse needs of individuals affected by these complex injuries.

Minimally Invasive Microsurgical Sealing of CSF Leaks 

A CSF loss occurs when a defect in the dura (cranial or spinal) allows the CSF to exit. Communication between the CNS, an immune-privileged compartment, and the rest of the body involves a risk of infection or intracranial hypotension due to decreased intrathecal fluid volume and subsequent downward traction on the brain. In 80% of cases, the cause of the loss is traumatic, and the typical locations of the cranial loss are the sphenoid sinus and the cribriform plate, which are in communication with the dura. The cause of the leaks could also be iatrogenic; this occurs in 16% of cases during cranial and spinal surgeries with a rate of 5.5-9% or due to lumbar punctures or epidural anesthesia. The phenomenon could also be spontaneous, but it is rarer, about five patients per year out of 100.00 in the United States, with peaks of age between the fourth and fifth decade of life [136,137]. The formation mechanism of these spontaneous spinal CSF discharges is still misunderstood. Still, they are probably due to structural weakness and thinning of the dura with a possible overlapping mechanical trauma and, in some cases, with an underlying genetic predisposition, as can occur in connective tissue syndromes, such as Klippel-Trenaunay syndrome. This syndrome is already associated with a wide variety of vascular and neurological abnormalities, such as venolymphatic malformations, and, in a study conducted in 2020, was also associated with a predisposition to spontaneous intracranial hypotension (SIH), found in four out of 67 patients [138]. Risk factors include female sex (female patients on average are about 60-65%), tissue disorders, degenerative diseases of the spine, trauma, or bariatric surgery [139]. The most adopted classification system for the spontaneous loss of spinal CSF (proposed by a study conducted by Schievink WI in 2016, based on 568 patients, of which 65.7% are women) groups CSF losses into four types according to the characteristics of the thecal sac defect. Dural tears cause type 1 leaks; they can be divided into 1a (ventral loss) and 1b (posterolateral loss). Type 1 leaks are commonly associated with pre-existing spinal diseases, such as dagger-like osteophytes and calcified disc herniation, often requiring surgical treatment and, if left untreated, can lead to permanent neurological deficits or spinal cord hernia. Type 2 losses are caused by diverticula in the meninges and are divided into simple (2a) and complex (2b). Type 3 leaks are venous fistulas of the CSF, and type 4 leaks are leaks that do not have an identified location. Without intervention, it has been noted that spinal CSF leakage progresses to debilitating neurological complications, including superficial siderosis, hearing loss, imbalance, visual disturbances, and brain fog [137].

How to Identify Leaks

Locating the exact site of a spinal CSF leak is necessary for a successful diagnosis and effective treatment, but this is only sometimes so simple with classic techniques. Recent advances in myelography techniques have improved leakage site detection and increased treatment options for patients with SIH. Several 2018, 2019, and 2020 studies have shown the best methods for identifying the exact loss point. In a study of 74 patients (from 2018), classical MRI and radiography showed no leakage in 14 patients. Still, a conventional dynamic CT myelography in the prone position or lateral decubitus identified a leak caused by a calcified microspure in 10 patients and a dural laceration under the root of a spinal nerve in the other four [140]. A 2019 study highlighted the correct positioning and the diagnostic imaging modality of choice for detecting CSF venous fistulas: digital subtraction myelography (DSM). In this study, 23 patients (16 women, with an average age of 52.5 years) who showed no evidence of CSF leakage on conventional spinal imaging underwent DSM. When performed in the lateral decubitus position, 74% of the cases were positive for CSF venous fistulas, compared to only 15% detected in the prone position [141]. For other CSF losses, a study conducted in 2020 on 62 patients observed the accuracy of conventional dynamic myelography (CDM), which correctly identified the loss in 61 patients [142]. This procedure has the highest temporal resolution and allows great flexibility in patient positioning. It also compares favorably with other dynamic examinations compared to the radiation dose and does not require general anesthesia. Thus, DSM and dynamic CT myelography are the diagnostic modalities of choice for defining the location of the leaks, which are very important factors for successful treatment.

Best Minimally Invasive Surgery Techniques

Several new surgical techniques allow the repair of these leaks; different ones are used depending on the type of leak. Several approaches to MIS have been used in recent years, such as transvenous endovascular embolization. This approach aims to get to the site of the leak, or the fistula, passing through a catheter through the Azygos vein or the plexus of the lumbar vein once the embolic agents (such as coils or liquid embolic) arrive and are distributed to seal the abnormal connection between the space that CSF and the venous system. This method can avoid open surgery and all problems related to it, such as infections, costs, operation time, tissue disruption, and, last but not the least, the recovery time for patients and the minimum incision, for example, the immediate closure of the postoperative skin after a minimally invasive multilevel laminectomy (three levels) is about 3-4 cm, which can be aesthetically pleasing for the patient [143]. Another approach used in a 2019 study was a tailored hemilaminectomy with intraoperative neurophysiological monitoring (IOM). In this way, they minimized bone removal and reduced tissue damage. At the end of the surgery, none of the 47 operated patients reported permanent neurological deficits, and only four had transient deficits [144]. In a more recent study (2022), five patients aged 44 to 62 years, of whom 60% were women, with six cerebrospinal-venous fistulas (CVFs), were operated on with a spinal CVF MIS ligation and none of them experienced surgical complications; one patient had two MIS procedures within three months, without complications and complaints, showing the benefit of this reduced invasiveness approach. 

The diagnostic imaging stage included an X-ray of the spine, MRI, different position DSM, and CT scan. During surgery, an IOM was used, and the site of the leaks was confirmed with a simple lateral radiography and after the incision and insertion of the tube with a fluoroscopy. All patients were discharged on the second day of postoperative surgery and seen six weeks later. None of them had complications or wound infections, complete resolution or improvement of symptoms was confirmed in patients, and even four returned to work within three months of surgery. The protocol included follow-up MRI, neurological status control, attendance, quality headaches, and, as we said, the ability to return to work. In addition, perioperative complications did not occur in any of the patients, and the success rate was 100%, higher than an open surgery epidural blood patch, with EBP (about 70%) [145]. 

In conclusion, MIS ligation of the CVF is safe and efficient; it is a less invasive procedure, reducing the risk of wound infections, the time of surgery, and re-recovery, as we have seen the patient could be discharged on the second postoperative day, significantly reducing maintenance costs and greatly increasing the comfort and well-being of the patient. There is a more recent study (2023) that compares other surgical approaches, such as ventral approaches, including cervical and thoracic discectomy or single/multilevel level corpectomy, or extended ventral approaches, including sternotomies and thoracotomies, with an MIS dorsal approach using a 2.5 cm skin incision to insert non-expandable tubular reactors, custom interlaminar fan extraction [146]. This method allows the surgeon to target specific spinal structures without extensive bone removal, minimizing soft tissue disruption and helping the patient's recovery. In this study, 58 patients, with an interval of 36-55 years, of whom 65.5% were female, were operated on with losses of type 1a, 1b, and 3 CSF. This approach reduced surgery time to 90-130 minutes, significantly lowered the rate of permanent neurological deficits to 1.7%, and achieved a success rate of 96.6% due only to the impossibility of identifying, in some cases, the correct location of the loss. This method showed less postoperative pain, and at a follow-up of 16-28 months, 45 out of 50 patients had reported better conditions. It was also discovered that with a dorsal approach, CSF leaks could be treated anywhere on the 360° circumference of the thecal sac, reducing the risk for patients [146]. 

Future perspectives and research

Further studies are needed to define the pathophysiology of SIH and the nature of spontaneous leaks of the CSF. The limits of these various studies are the retrospective design, the single-center approach, and the minimal number of patients; in conclusion, further studies with a higher number of patients are needed mainly to evaluate the detailed long-term surgical outcome. The data are encouraging for now, as these procedures shorten hospital stays, reduce costs, minimize clinical complications, and significantly enhance patient satisfaction.

Table 2 summarizes and compares the different minimally invasive spine surgery techniques mentioned above with open surgery.

**Table 2:** Summary of the newest minimally invasive neurosurgery techniques for spine surgery

Techniques	Applications	Advantages	Risks	Outcome vs, open surgery
Endoscopic keyhole for spinal surgery	• degenerative spine diseases • disc herniations • spinal tumors	• less tissue damage • excellent visualization	• limited workspace • technical complexity	• faster recovery
Percutaneous endoscopic lumbar discectomy (PELD)	• lumbar and cervical disc herniations • infectious spondylodiscitis	• reduced surgical trauma • reduced blood loss • avoided risks of general anesthesia	• dural tears • CSF leaks • nerve root injuries	• faster recovery • similar/lower recurrence rates • less post-operative pain
Lateral lumbar interbody fusion (LLIF)	• spinal deformities • degenerative disc diseases	• biomechanical correction with minimal muscle trauma	• transient lumbar plexus irritation • limited to L5-S1	• faster recovery • improved long-term stability • fusion rate >90%
Percutaneous vertebroplasty and kyphoplasty	• osteoporotic vertebral fractures	• immediate vertebral stabilization • reduced risk of leakage	• adjacent vertebral fractures	• faster recovery • reduced long-term medications
MRI-guided cryoablation	• metastatic spinal tumors	• precise targeting • real-time imaging	• limited workspace • neural injury	• faster recovery • lower complication rates
Robot-assisted techniques	• complex spinal tumors	• high precision • less tissue damage	• high costs • system errors • specialized training	• faster recovery
Robotic-assisted pedicle screw placement	• spine stabilization • deformities or fractures	• reduced radiation exposure • accurate screw placement	• high costs • system errors • specialized training	• faster recovery • lower complication rates

Early Minimally Invasive Removal of Intracerebral Hemorrhage (ICH) 

ICH is a blood extravasation into the brain parenchyma; in the past, it was commonly referred to as "hypertensive hemorrhage," but hypertension is, in many cases, considered as the etiology. ICH is the second most common form of stroke (15-30% of all types of strokes), about 12-15 cases per 100,000 per year, and the most deadly. ICH has an initial hospitalization mortality rate of 30-50%, a one-year survival rate of 50%, and a five-year survival rate of 30%. Among survivors, only 33% are independent at work within six months of bleeding. Early studies showed an incidence equal to subarachnoid hemorrhage, SAH, but more recent studies in the CT era showed at least twice the incidence of SAH. The onset is usually during activity, rarely overnight, and is probably related to increased blood pressure, BP, or increased cerebral blood flow, or CBF. According to some studies, ICH is classified in its primary or secondary causes, respectively, with a rate of 80/85% and 15/20%. Primary ICHs are more than 50% closely related to hypertension as a risk factor, and 30% are associated with cerebral amyloid angiopathy (CAA). 

On the other hand, the causes of secondary ICHs include conversion of ischemic stroke hemorrhage, stimulant drugs, vascular malformations, such as aneurysms, AVMs, venous angioma, cavernoma and dural arteriovenous fistula, neoplasms, trauma, vasculitis, Moyamoya's disease, or sinus venous thrombosis. Another classification used in clinical practice distinguishes between deep and lobar ICHs based on location. Deep ICHs are found in the basal ganglia; the putamen is the most common, 50%; thalamus 15%, pons 10-15%, cerebellum 10%; brain white substance 10-20% or brain stem 6% and are generally related to hypertension. Lobar ICHs usually require more in-depth diagnostic tests due to their expansive range of causes. There are many risk factors: age, as incidence increases a lot after 55 years and doubles with each decade of age up to the age of > 80 years, where it is 25 times that during the previous decade; gender is more common in men; race; and living in the United States. ICH affects Black individuals more than White individuals, probably due to the higher prevalence of hypertension in the former. Other risk factors are any previous strokes of any kind, chronic alcohol consumption (a study suggests that consuming >3 drinks (12 g of EtOH) per day increases the risk of ICH by seven times), atrial fibrillation, diabetes mellitus, and CNS infections such as herpes simplex encephalitis (which produce low-density lesions, progressing into bleeding or even coagulation disorders) [136]. 

A 2021 study with a large cohort of 113,430 patients analyzed various clinical results defining the various complications: ICH is commonly characterized by cerebral edema in most patients, hematoma dilation, hydrocephalus, pneumonia, infections, epilepsy, convulsions, venous thromboembolic events, and early neurological deterioration within the first hours of onset. These conditions have increased the mortality rate and costs and prolonged hospital stays of up to 12 days, with hydrocephalus as the deadliest complication. Hence, there is a need for early treatment [147]. The most common complication during an ICH is an expansion of the hematoma (HE), which must be at least 33% or 6 ml to be defined as such. However, these thresholds could be more precise because, as seen in a study conducted in 2023 on 987 patients with spontaneous ICH, there is a strong interaction between the increase in relative and absolute volume and the localization of ICH. This interaction could significantly predict discharge and the 90-day modified Rankin scale (mRS) scores [148]. The mRS is a scale used to measure the degree of disability or dependence in a patient's daily activities, especially after a stroke or ICH. The scale varies from 0 to 5: 0 is no symptoms. A scale of 1 is no significant disability despite the symptoms; the patient can perform all the usual activities and duties. A scale of 2 is mild disability; the patient can take care of his business without assistance but is not able to carry out all the normal activities and responsibilities. A scale of 3 is moderate disability; the patient needs a little help but can walk without assistance. A scale of 4 is moderately severe disability; the patient cannot meet his physical needs without help and cannot walk without assistance. A scale of 5 is severe disability; the patient is bedridden and incontinent and requires constant nursing care and attention [136]. 

The 90-day mRS scores refer to the disability assessment using the mRS conducted 90 days after stroke or ICH. This study found that the optimal cutoff points for volume increase for the worst mRS loss were 0.86 ml or 5.1% in depth and 6.91 ml or 19.2% in lobar ICH, and for the worst 90 days, mRS was 9.66 ml or 9.3% in deep and 26.64 ml or 25% in ICH lobar. In conclusion, the effect of HE on the result is modified by location; deep structures have a lower tolerance for HE than lobar ones. Another study showed that HE can be accurately predicted by non-contrast CT and NCCT radiographic characteristics using a transport-based morphometry TBM algorithm. In these cases, using the technologies could significantly improve the clinical outcome for patients [148]. ICH could also be traumatic (tICH), and as seen in a study conducted in 2022 and presented at the annual AANS meeting, there is a clinical association between tICH and cerebral vasospasm. The study identifies various risk factors in patients with tICH that increase the likelihood of developing vasospasm, such as advanced age, Glasgow coma scale, GCS score <9, intraventricular hemorrhage IVH, smoking, cocaine use, fever, and hypokalaemia. Vasospasm in tICH patients is associated with a more complex and expensive hospital course, including a lower probability of routine discharge and prolonged hospitalization. However, mortality does not appear to increase [149]. 

Recommended Neuroimaging

CT and NCCT are more relevant for an acute stroke. CTA, enhanced by contrast, is instead able to identify patients at high risk of bleeding extension; MRI is better for diagnosing ICH, small vessel disease, vascular abnormalities, and amyloid angiopathy; and catheter angiogram is better for diagnosing aneurysms. A 2019 study suggested a diagnostic flowchart that incorporates size, location, clinical risk factors, and pathogenesis that, when considered together, can lead to a more accurate diagnosis and, therefore, to better manage ICH in cases of acute stroke. Starting with NCCT and a CTA could help identify ICH and the spot sign and evaluate vascular abnormalities. NCCT and CTA show subarachnoid hemorrhage, SAH, unusual hematoma configuration, or suspicious imaging. In that case, an MRI should be performed, which helps diagnose ICH and assess small vessel disease, amyloid angiopathy, and vascular abnormalities. If the MRI is negative, it is better to perform an angiography with the catheter to check for the presence of AVMs or aneurysms. Depending on the patient's condition, potential follow-up could include repeated angiograms, additional MRI, or long-term monitoring [150].

Clinical Trials and Minimally Invasive Techniques

A recent study (2024) in Switzerland analyzed and compared the minimally invasive approach to the gold standard, the best medical treatment, BMT, as a combination of medical blood pressure control, intensive care and prevention of secondary complications, and evacuation of open surgical hematoma [151]. Both these treatments have low rates of good functional outcomes and patient survival. The study, MISTIE, MIS plus rtPA for the evacuation of ICH, evaluated the potential superiority of the MI approach using an SA (stereotactic insertion of a catheter into the hematoma cavity, repetitive irrigation of the blood clot using thrombolytic agents, and sequential hematoma drainage). MISTIE II effectively reduces hematoma volume, clinical safety, and feasibility. MISTIE III evaluated a good functional outcome after one year (mRS ≤ 3 points), showing no significant difference between SA and BMT. At the same time, SA significantly reduced all-cause mortality throughout the study period. 

A subgroup analysis of MISTIE III also shows that the patient who underwent surgery within 36 hours of the onset of symptoms had a better outcome than the others; it was seen that the delayed start of treatment reduces the rate of better results by 5% per hour. The surgery was planned with a neuronal navigation system to define the best entry point, after which a burr hole was made at the entry site, and a transparent trocar was inserted as a working channel for the endoscope. Continuous aspiration and irrigation are used to aspirate the hematoma under direct visualization, with endoscopic coagulation and hemostatic agents controlling bleeding areas. After removal, a postoperative CT was performed to verify the completeness of the evacuation; otherwise, a re-aspiration was conducted. In conclusion, the study assessed that the evacuation of a sICH (in this case, spontaneous supratentorial ICH, SSICH) in an endoscopic minimally invasive approach within six to 24 hours and achieving a rapid and significant reduction in hematoma volume will result in better outcomes and a lower morbidity rate than BMT. Therefore, the survival rate associated with MIS is lower, mainly when performed within the recommended period, and patients who have undergone endoscopic MIS report higher satisfaction and quality of life scores than those receiving BMT due to faster recovery and fewer complications. 

A 2021 Swedish study proposed a SwICH surgical score in patients treated with SSICH based on several patients treated from 2009 to 2019 in a single center [152]. The 126 points had an overall 30-day mortality rate of 23%; all points with a score of 0 survived over a year. This new score was a good predictor of 30-day mortality but did not outperform the previously established ICH score in predicting 30-day mortality. A more recent (2024) TRIAL was designed to evaluate minimally invasive trans-sulcal parafascicular surgery (MIPS) approach. The study included 300 patients randomly assigned in a 1:1 ratio in a surgical group; the surgery was performed with a minimal BrainPath access port that allowed the bimanual technique with visualization and a Myriad device used to evacuate with aspiration hematoma (an approach used in 2022, feasible and technically practical) or in a control group, treated with guideline-based medical management alone [153]. Among the patients, 30.7% had anterior basal ganglia ICH, and 69.3% had lobar hemorrhages, with a hematoma volume of 30 to 80 ml and a GCS between 5 and 14. The percentage of patients who died within 30 days was 9.3% in the surgical group and 18% in the control group; five patients in the surgery group had postoperative bleeding and neurological deterioration. Among patients who could be performed surgery within 24 hours after an acute hematoma evacuation, ICH MIS resulted in better outcomes at 180 days than those with guideline-based medical management and also achieved a significant reduction in hematoma volume (73.2%), preventing secondary brain injuries and improving short- and long-term outcomes. The problems of the study were mainly the narrow population analyzed and the selection of patients. Since the results vary depending on the location of the bleeding, surgical intervention was more beneficial for lobar hemorrhages than the basal ganglia. 

A study conducted in 2023 in New York showed that evacuation time was significant; when surgery was performed within 12 hours of stroke, the probability of functional independence of six months, equal to or less than 2 (according to mRS scores), was 40.9%; when it was conducted from 12 to 24 hours 33.3% and 24 to 48 hours 31%, after 48 hours, the probability dropped to 4.5%. Thus, an evacuation conducted within 24 hours was associated with a 17.7 times greater likelihood of achieving long-term independence. However, this study found that patients with early evacuation (<24 hours) had even lower rates of anticoagulant drugs and mortality rates but a higher probability (82% vs. 45%) of encountering intraoperative bleeding and a longer surgery time [154]. The following year, in 2024, a five-point scale was presented and applied to the severity of the bleeding found; a score of 1 indicated no bleeding; 2, minimal bleeding; 3, bleeding that requires cauterization; 4, bleeding that requires cauterization for at least 15 m to reach hemostasis; and 5, bleeding that requires cauterization for at least one hour. In 142 patients in the study, the mean bleeding score was 2 [155]. Increased preoperative volume, concomitant intraventricular hemorrhage, and early evacuation time were associated with increased bleeding scores, as included in the NY study, especially the ultra-early ones, associated with bleeding scores 2.4 points higher than the subsequent evacuation. However, despite higher intraoperative bleeding scores, patients undergoing ultra-early evacuation, <5 hours, did not have a higher probability of postoperative bleeding or, worse, mean mRS scores of six months. In conclusion, despite the higher IO bleeding, a minimally invasive endoscopic technique with large IO visualization, active irrigation for targeted tamponade, and direct cauterization would improve patient outcome, median mRS scores of six months, and mortality rate.

Limitations and Actual Challenges

All these studies have limitations, including restricted populations and retrospective analysis, usually of a surgical cohort of a single institution; large randomized controlled trials are required to determine effective short-and long-term functional outcomes. A recent study in swine showed that local application of controlled subatmospheric pressure to an ICH can safely remove more than half of the clot in one week and more than 90% in two weeks without evidence of bleeding. This mechanical tissue resuscitation technique, MTR, could be evaluated and studied better [156].

Economic and healthcare system impact

Cost-Effectiveness of Minimally Invasive Procedures 

Minimally invasive surgeries offer many benefits, one of which is enhanced precision. Precise instrument control has many added benefits, such as reduced blood loss, smaller incision size, shorter in-hospital stay, lower infection risk, and minimum post-operative pain. In addition, these techniques offer rapid patient recovery and minimal postoperative complications. Patients approached minimally are observed to get discharged earlier. Minimally invasive procedures are associated with significantly shorter hospital stays than open traditional surgeries. As a result, reducing the total length of hospital stay reduces inpatient care costs. Spine surgeons were one of the first neurosurgical subspecialties to adopt cost-effectiveness. This is because of high procedure costs and pressure from the insurance companies to justify treatment protocols. Indirect costs play a significant role than direct costs in determining the overall cost-effectiveness of minimally invasive spine surgeries [157]. 

In a study by Parker et al., MISS saved approximately $8731 on average per patient, with indirect costs contributing nearly three times more than direct savings [158]. A study by Udeh et al. showed that MISS was more cost-effective than an open laminectomy [159]. In addition, a survey by Band et al. proves that minimally invasive lumbar infusion surgery reduces the length of hospital stay and inpatient narcotic use [160]. Patients who had undergone minimally invasive lumbar infusion surgery stayed in the hospital for a significantly shorter time (1.6 days vs. 2.4 days) and had reduced need for extra narcotics and fewer opioids during their hospital stay (51 mg vs. 320 mg) [159]. However, according to Zygoraukis et al., minimally invasive procedures and open surgeries are equally cost-effective, meaning that neither of the two techniques supersede in terms of cost-effectiveness [157]. Patients experience fewer complications, such as reduced blood loss and lower post-surgical infection risk. 

In a study by Patel et al., patients who underwent open posterolateral lumbar fusion (313 ccs) and open posterior lumbar interbody fusion (514 ccs) were associated with significantly higher blood loss than patients treated with the minimally invasive technique (316 ccs). In addition, patients with open PLF (163 cc) received blood transfusion products more frequently than the MIS TLIF group (6 cc). Red blood cell products were transfused in 29% of open PLF cases and 42.9% in the open PLIF/PLF group. However, blood transfusion was rarely required in the MIS TLIF group. Additional costs associated with blood transfusions should be taken into account. A study by Patel et al. mentions that replacing one unit of donated blood costs around $250 [161]. In addition, minimally invasive procedures carry minimal risk of surgical site infections and have lower infection rates compared to open surgeries. 

Traditional open surgeries lead to higher infection rates, significantly increasing costs because of prolonged hospital stays, additional diagnostics, and treatment regimens [162]. Minimally invasive procedures allow neurosurgeons to focus on intricate details with high accuracy, optimizing surgical outcomes while minimizing tissue damage. Such techniques result in smaller incisions when compared with open surgeries. This has several patient benefits, including reduced scarring and less post-operative pain. Thus, patients experience less post-surgical discomfort. In addition, patients enjoy the cosmetic advantages of smaller incisions. In addition, due to smaller incision sizes and constrained exposure to external contaminants, minimally invasive techniques save patients from deadly microorganisms [163]. A study by Clark et al. reports shorter surgical time in patients undergoing percutaneous cervical laminoforaminotomy (115 minutes) than the open group (177 minutes) by a 62-minute difference [164]. When compared with open traditional surgeries, minimally invasive procedures often lead to shorter in-hospital stays. In a study by Platt et al., patients who had undergone open posterior cervical foraminotomy stayed, on average, 58,6 to 304,8 hours in the hospital, whereas the MIS group stayed for only 20 to 273,6 hours [165]. Therefore, minimally invasive procedures lead to shorter surgical duration, decreased need for blood transfusions, lower infection risk, reduced need for opioids or other symptomatic medications, lower pain levels, reduced hospital stay, and higher satisfaction from patients with quicker recovery and earlier mobilization.

Healthcare System Impact 

Patients treated with minimally invasive procedures experience lower blood transfusion rates, reduced risk of infection, less pain, and faster recovery. Such factors add to shorter postoperative in-hospital stays, saving abundant healthcare resources and freeing up beds and healthcare professionals for other needy patients [163]. Minimally invasive procedures offer lower compilation rates. Fewer complications translate to decreased healthcare costs related to hospital readmissions and follow-up surgeries. In the long run, minimally invasive procedures save readmission costs by reducing complication rates that contribute to improved patient recovery. Minimally invasive procedures lead to lower reoperation rates in comparison with open surgeries. In a study by Altshuler et al., the reoperation rate was 10.4% in MIS patients and 12.2% in patients treated with open traditional methods. In addition, patients who underwent MINPs were comparatively less likely to enroll at a rehabilitation facility than patients who had undergone conventional open surgeries [166]. 

MINPs such as robotics help enhance physicians' surgical skills, making them eligible to perform complex yet intricate operations with high precision and control. Such techniques challenge a surgeon's hand dexterity and intuitiveness, forcing them to refine their skills and deal with tasks more efficiently. In addition, fine instrument control and high-definition 3D visualization improve surgical outcomes as they help expand minute structures on the big screen. This ultimately benefits the patients by reducing the risk of mishaps and postoperative complications. To ensure safe surgical proficiency, comprehensive training programs are organized for surgeons interested in expanding their skills. These programs offer hands-on experience with excellent mentorship opportunities. Simulation exercises based on real case scenarios help prepare surgeons for the operation day [167]. In conclusion, minimally invasive neurosurgical techniques have wide-ranging implications for health systems, as they reduce resource consumption, improve surgical training, and lower long-term healthcare costs. Through fewer readmissions and less necessity for rehabilitation, minimally invasive neurosurgical procedures have significantly improved patient outcomes while representing a more cost-effective neurological surgery strategy. Indeed, minimally invasive neurosurgical procedures have evolved from a novel approach to a standardized practice. These benefits can only be maximized to their full potential through continued investment and research in surgical technology and training.

Technological limitations 

Many complications and technical issues are raised as science discovers more techniques to reduce invasiveness. Pre-existing cavities are used to access the target site, limiting how to access the surgical site. Over the last four decades, advancements in neurosurgical technologies have revolutionized the concept of surgery. The shift from open surgery to a keyhole transoral approach is a passageway to extremely minimally invasive surgeries. With a cascade of development, technology changed concerning the type of approach and common complications; with the arising complications, it was necessary to have a three-dimensional planning system to analyze the surgery intraoperatively. There are many technical limitations encountered in minimally invasive neurosurgeries that will be addressed later. Following numerous tests using endoscopes from different manufacturers on various corpses, it was determined that improvements were necessary to handle the problems and difficulties that arose. The majority of the instruments were found to be incompatible with the stereotactic system. They could not accommodate the brain's anatomical features; for instance, an endoscope with an 8 mm diameter could not pass through the foramen of Monro because its diameter was less than 6 mm. When the endoscope and video camera were connected, the investigator's hand had a negative center of gravity behind it. The optical and light bundles escaping the cable made it worse. 

In a research published by F. Duffner et al. [168], a new endoscope system was designed and manufactured by Schölly Co. (Denzlingen, Germany) for CRW (Cosman-Roberts-Wells). The endoscope consists of a 2.30m length and 20000 fibers combined. The visual results of the endoscope are close to the rod-lens system, and an improvement in results is seen over the traditional use of optic bundles in fiberscopes. The 2.30m length of the endoscope allows it to be placed in a non-sterile area in operating wards. The optical and light bundles are positioned in a single cable. As the camera is no longer connected from behind the surgeon's hand, this decreases the unfavorable effect of gravity. The system has a standard camera mounting; thus, the same camera can also be used for other purposes. This saves the space and makes the setup less complicated and more efficient. Such techniques can be used in endoscopically guided surgeries with open MRI. Microdrive is added to the distal end of the guiding cannula. This overcomes the distance to the target, where access is limited. A range of 3 cm is added to the endoscope just by adding a Microdrive. In collaboration with Schölly Co., a multipurpose catheter has been created. The catheter can reach deep-seated tissues and targets because of its 2 mm diameter. Its 0.99 mm diameter working channel and 1 cm flexible segment at the tip enable control of flexible equipment like coagulation probes and balloon catheters. A baby endoscope can be guided using a catheter. 

Compared to standard catheters, the multifunctional catheter has advantages. The procedure is safer because the catheter serves as a permanent visual guide for the procedure, increasing the accuracy of endoscopic procedures without requiring the endoscope to be moved. Single-use coagulation probes are used together with multifunctional catheters. Cutting and coagulation are made possible by the adjustable wire in the middle of the tip, which shifts the wire screw's position. Bipolar punch, which enables coagulation and tissue grabbing in a single phase, results from further advancements. When this method was tested, obstacles were overcome, and expectations were met. For laparoscopic and minimally invasive neurosurgeries, monopolar or bipolar electrodes, or even the smallest flexible forceps and scissors, can be placed through the trocar's channels. The latest generation of micro instruments has a distinctive and straightforward design. According to a study by J. Wahrburg, new methods for MIS are being thoroughly researched and assessed [169]. There are still some challenges in applying these techniques as there are fewer natural paths in the human body to follow. Such restrictions can be prevented by stereotactic planning and mounting rigid endoscopes on a stereotactic frame or a newer, frameless mechanical system. Improvements are required mainly in instrument flexibility and increasing functionality, giving full access to the surgeon.

Potential for future research 

The advancements over the last decade have made it possible to perform surgery without directly accessing the surgical site. The revolution from open surgery to minimally invasive surgeries through an endoscope is a pivotal point that will alter the current practice. With ongoing research, technology will transform the surgery to be more minimal and less invasive, and soon, it will allow the surgery to be conducted beyond the set parameters of traditional surgery. Surgery is transforming from a laparoscopic approach to a transoral approach. The development of medical devices, robotics, AR, and VR approaches integrated into surgery will aid in reaching deep tissue, delivering sutures, energy for ablation, and excising the necrotic tissue [168]. Minimally invasive techniques in neurosurgery have taken multiple ways towards development, using an endoscope, frame-based stereotaxy, keyhole approach, and neuronavigation. Minimizing approaches use pre-existing cavities and spaces to approach the surgical site. Although rapidly new methods are coming to light, many problems still need to be solved. Often, unusual scenarios are witnessed in surgeries that are away from usual anatomy or pathologies; this creates many complications that need to be studied [168]. 

Recent advancements in nanotechnology have signaled extremely minimal invasions to surgical sites. It has enabled broader visualization at deeper levels and more efficient drug delivery. Although there are unique challenges in the field of nanotechnology in neuroscience, it has a great potential to enhance results in a neurosurgical review written by Mattei et al. The small size of nanoparticles and nanotools were studied. The size is significant as it reduces invasiveness and increases therapeutic interventions. Previous studies have demonstrated a wide variety of nanotechnology applications in MINS and have great potential in advancing neurosurgical procedures in the future [170]. These nanotechnologies work as imaging techniques using Quantum dots and metallic nanocrystals; different color emissions are seen when excited with a single UV lamp. These can be controlled and manipulated systematically. Non-electrochemical systems (NEMS) and microelectromechanical systems (MEMS) are devices manufactured at nano levels and composed of refined miniaturized electrical and mechanical apparatuses. Further development of nano knives has demonstrated the dissection of individual axons on peripheral nerves in vivo mouse models [171]. According to the currently available literature and research, nanotechnology may overcome major hindrances in complicated surgeries and reduce invasiveness. Certain diseases with unclear pathologies may also be treated using nanotechnologies and imaging techniques. Some biocompatibility issues still need to be addressed regarding the use of nanotechnology in MINS. Still, these difficulties can be overcome with several strategies and the incorporation of biopolymers. Neurosurgeons and scientists must collaborate and clinically study the efficacy, toxicity, biocompatibility, and cost-effectiveness. 

By integrating nanotechnology into routine neurosurgical procedures, a reduced rate of mortality and an increased rate of successful surgeries may be seen. With the integration of supercomputers and AI, accurate pre-judgments can be made to avoid future complications [170]. AI can help detect neoplasia biomarkers, and with advancements, it will be easier to predict accurately before the onset of the disease. Nanotechnology and nanorobotics will enhance the preoperative, intraoperative, and postoperative imaging of the targeted organs. Moreover, it can signal the safest route to access the site without disturbing the surrounding organs. In the future, as the AI trains more, the accuracy will be enhanced closer to 100 percent, and it may help fully eradicate the disease without the least complications and invasiveness. Minimally invasive techniques are expected to transform into less invasive techniques, reducing the surgical corridor and widening the visuals. Moreover, including robotics may contribute to a successful rate of complicated surgeries while maintaining the surgeon's autonomy. These surgeries will be performed globally with robotics, and the control center in some other part of the world can perform them without the surgeon being physically present. As the research is carried out, many changes will be implemented to alter the current practice.

Anticipated technological developments 

Healthcare is transforming due to technologies like 5G, AI, and robotics, particularly in the planning and execution of surgeries. Advancements are enhancing the precision, accessibility, and efficiency of brain and spine surgeries. Future surgical innovation relies on 5G networks, facilitating real-time remote procedures with exceptional speed and low latency. This skill enables surgeons to swiftly perform complex procedures in remote or underserved regions, enhancing global access to specialized care [172]. AI and ML facilitate selecting optimal surgical procedures and outcome prediction by analyzing extensive datasets. These technologies enhance surgical decision-making, thereby increasing the efficacy of procedures. Robotic-AI hybrid surgical systems are transformative. In intricate brain and spinal cord surgeries, real-time AI-assisted robotic surgical arms enhance precision and minimize errors. Neurotechnologies such as brain-computer interfaces (BCIs), deep brain stimulation (DBS), and spinal cord stimulation demonstrate the interaction between technology and the nervous system to enhance neurological functions, offering hope to affected patients [173]. 

Recent developments in radiology encompass integrating AI technologies in the spine and spinal cord imaging. Automated segmentation, lesion detection, and bone density assessments improve diagnostic precision and patient management. These advancements allow healthcare practitioners to detect issues early, enhance predictive accuracy, and streamline workflows. Advanced imaging techniques and AI algorithms are improving personalized treatment options via diffusion tensor imaging (DTI) and AI-assisted lesion characterization [174]. These technologies exhibit considerable potential while simultaneously eliciting ethical and social concerns. Data security, patient privacy protection, and equitable access promotion are critical priorities. Trust in transparent and explainable AI systems is crucial for clinicians and patients, requiring the establishment of equitable implementation frameworks to address socioeconomic disparities in access to these technologies [175]. AI, robotics, and 5G networks drive healthcare innovation. These technologies enhance surgical precision and broaden access to advanced medical treatments. These advancements may transform healthcare delivery by integrating technical and clinical applications, providing previously unattainable solutions. The future of medicine relies on the successful and equitable integration of these tools into clinical practice through collaboration among physicians, engineers, and ethicists.

## Conclusions

Advanced technologies including AI, AR, and robotics have transformed neurosurgical and cancer treatments through minimally invasive procedures. AI models such as Swin-Unet achieve segmentation accuracy of 93% while minimally invasive procedures deliver success rates above 96%, shorter recovery times, and fewer complications, which preserves healthy tissues. Combining for spinal surgery, transforaminal approaches with percutaneous lumbar endoscopic discectomy, dynamic myelography, and tubular retractors show significant advantages in treating complex cases, such as CSF loss. The combination of traditional surgical methods with advanced tools through hybrid strategies creates a patient-centered care model that delivers safer and more effective treatments with reduced invasiveness. The immediate requirement is essential research and multidisciplinary collaboration to guarantee safe implementation of these innovations which must also provide fair access to patients who will ultimately benefit from long-term advantages.
